# RETSAT associates with DDX39B to promote fork restarting and resistance to gemcitabine based chemotherapy in pancreatic ductal adenocarcinoma

**DOI:** 10.1186/s13046-022-02490-3

**Published:** 2022-09-15

**Authors:** Qiu Tu, Xiuyun Liu, Xiaoqing Yao, Ruixue Li, Gaojing Liu, Honglv Jiang, Kaiqin Li, Qiongfang Chen, Xiaoyan Huang, Qing Chang, Guoqiang Xu, Hong Zhu, Peng Shi, Bo Zhao

**Affiliations:** 1 Kunming Institute of Zoology, Chinese Academy of Sciences, Kunming, China; 2 Key Laboratory of Animal Models and Human Disease Mechanisms of Yunnan Province, Kunming Institute of Zoology, Chinese Academy of Sciences, Kunming, China; 3 University of Chinese Academy of Sciences, Beijing, China; 4 State Key Laboratory of Genetic Resources and Evolution, Kunming Institute of Zoology, Chinese Academy of Sciences, Kunming, China; 5 Center for Excellence in Animal Evolution and Genetics, Chinese Academy of Sciences, Kunming, China; 6 Department of Hepatobiliary and Pancreatic Surgery, Second Affiliated Hospital of Kunming Medical University, Kunming, China; 7 Jiangsu Key Laboratory of Neuropsychiatric Diseases and College of Pharmaceutical Sciences, Jiangsu Key Laboratory of Preventive and Translational Medicine for Geriatric Diseases, Soochow University, Soochow, China; 8 Primate Facility, National Research Facility for Phenotypic & Genetic Analysis of Model Animals, and National Resource Center for Non-Human Primates, Kunming Institute of Zoology, Chinese Academy of Sciences, Kunming, China

**Keywords:** *RETSAT*, *DDX39B*, Fork restarting, Hypoxia, Gemcitabine, Resistance

## Abstract

**Background:**

Severe hypoxia is a prominent character of pancreatic ductal adenocarcinoma (PDAC) microenvironment. In the process of gemcitabine based chemotherapy, PDAC cells are insulted from replication stresses co-induced by hypoxia and gemcitabine. However, PDAC cells get outstanding abilities to resist to such harsh conditions and keep proliferating, causing a major obstacle for current therapy. *RETSAT* (Retinol Saturase) is defined as a hypoxia convergent gene recently, with high expression in PDAC hypoxic sectors. This study aimed to explore the roles of *RETSAT* in replication stress resistance and hypoxia adaptation in PDAC cells, and decipher the underlying mechanism.

**Methods:**

The expression of RETSAT was examined in TCGA (The Cancer Genome Atlas), human pancreatic cancer microarray, clinical specimens and cell lines. Functions of *RETSAT* were studied by means of DNA fiber assay and comet assay in monolayer cultured PDAC cell lines, three dimensional spheroids, patient derived organoids and cell derived xenograft mouse models. Mechanism was investigated by using iPOND (isolate proteins on nascent DNA) combined with mass spectrometry, immunoprecipitation and immunoblotting.

**Results:**

First, we found the converse relationship of *RETSAT* expression and PDAC chemotherapy. That is, PDAC patients with high *RETSAT* expression correlated with poor survival, while ones holding low *RETSAT* expression were benefitted more in Gemcitabine based chemotherapy. Second, we identified RETSAT as a novel replication fork associated protein. HIF-1α signaling promotes RETSAT expression under hypoxia. Functionally, RETSAT promoted fork restarting under replication stress and maintained genomic stability. Third, we uncovered the interaction of RETSAT and R-loop unwinding helicase DDX39B. RETSAT detained DDX39B on forks to resolve R-loops, through which avoided fork damage and CHK1 initiated apoptosis. Targeting DDX39B using chemical CCT018159 sensitized PDAC cells and organoids to gemcitabine induced apoptosis, highlighting the synergetic application of CCT018159 and gemcitabine in PDAC chemotherapy.

**Conclusions:**

This study identified RETSAT as a novel replication fork protein, which functions through interacting with DDX39B mediated R-loop clearance to promote fork restarting, leading to cellular resistance to replication stresses co-induced by tumor environmental hypoxia and gemcitabine in pancreatic ductal adenocarcinoma.

**Supplementary Information:**

The online version contains supplementary material available at 10.1186/s13046-022-02490-3.

## Background

Severe hypoxia is a common character of pancreatic ductal adenocarcinoma (PDAC). Different from other solid tumors, PDAC consists of dense stromal fibroblasts and inflammatory cells, with abnormal or absent vascularization in central sectors, resulting in over-desmoplasia and quite limited oxygen diffusion through the tumor [1], with median 0.3% oxygen in its tumor microenvironment (TME) [2]. Indeed, TME hypoxia-based therapeutic strategies have been studied and developed over years [3]. The adaptive mechanisms of PDAC cells to hypoxia has been deciphered regarding multiple aspects such as metabolic reprogramming [4, 5], redox homeostasis [6], stemness maintenance [7] and angiogenesis [8]. Many antagonists / agonists of HIF pathway and prodrugs targeting TME hypoxia have been developed [9], and some of them showed ideal therapeutic effects in both xenograft animal models and pre-clinical evaluation [10]. However, the PDAC clinical therapy is still regrettable, with 90% PDAC exhibits resistance to gemcitabine-based therapy, which is the first-line drug for PDAC treatment [11, 12], and 74% relapse post treatment [13, 14]. Thus, it is a major unmet clinical need to understand how PDAC cells are resistant to TME hypoxia.

Sufficient oxygen supply is necessary to DNA synthesis. Ribonucleotide reductase (RNR) is an enzyme consisting of two homodimeric subunits, RRM1 and RRM2 or RRMB2. RNR acts for dNTPs biosynthesis. The β subunit encoded by RRM2 or RRMB2 contains an oxygen-requiring di-iron tyrosyl radical site essential for catalysis [15]. Severe hypoxia challenges the activity of RRM2 β subunit and dNTPs level, which further induces replication stress [16]. One mechanism has been revealed that cells switch RRM1/RRM2 to RRM1/RRM2B enzyme under hypoxia in order to retain activity and preserve ongoing replication, even with much lower fork velocity [17]. Notably, gemcitabine blocks the catalytic domain of RNR to destroy dNTP pool [11, 12], leading to inhibition of DNA synthesis and cell cycle progression [18].

Apart from these exogenous threats, RNA-DNA hybrid named R-loop is a major obstacle in replication fork progression endogenously [19]. R-loop is formed during mRNA transcription and exists throughout the whole genomes. Especially, there are frequent collisions occurred between replication forks and transcriptional machinery in fast proliferating cells. Indeed, a few cleaners are working over the whole genome to remove R-loop structures in order to orchestrate DNA replication and transcription [20]. For example, RNase H1 is able to digest RNA component of R-loop [21], while DDX39B functions as a resolvase to unwind R-loop structures [22]. Persistence replication stress initiates ATR-CHK1 signaling to arrest cell cycle for DNA repair, or launches apoptosis if damage overwhelmed. Alternatively, cells use tolerant mechanisms to adapt to replication stress either through dormant origin firing [23], or through restarting replication downstream of the lesion and leaving behind an ssDNA gap [24]. This means in the process of gemcitabine based chemotherapy, PDAC cells face with replication stresses not only from gemcitabine toxicity and TME hypoxia exogenously, but also from R-loop endogenously. On one hand, PDAC cells must keep DNA synthesis for cell proliferation. On the other hand, they have to protect from fork damage and ATR-CHK1 signaling initiated apoptosis challenged by such harsh conditions [25]. The molecular mechanism underlying this paradox remains to be elucidated.

RETSAT (official name: all trans retinol 13,14 reductase) is an oxidoreductase with conserved protein sequence and genic organization between human and rodent homologs [26]. It plays roles in endoplasmic reticulum (ER) in cytoplasm to transform retinol into 13,14-dihydroretinol. PPARα in liver [27], PPARγ in adipose tissue [28] and FOXO1 in primary hepatocytes [29] function as upstream regulators of RETSAT expression. However, recent studies indicate that its functions might be more than its name suggested [30]. For instance, RETSAT protects fibroblasts from ultra violet (UV) or paraquat induced oxidative stress [31], indicating unknown functions of RETSAT in oxidative homeostasis, or even UV induced DNA damage response and genomic stability. Using evolutionary genome comparison, we identified *RETSAT* to be a convergent gene in mammalian adaptation to hypoxia on the Qinghai-Tibetan Plateau, and the amino acid switch from glutamine (Q) to arginine (R) at the position 247 (Q247R) of RETSAT is responsible for heart function enhancement and mammalian adaptation to hypoxia [32]. *RETSAT* mutation is correlated to occurrence of undifferentiated tongue sarcoma [33], and its expression is positively associated with tumor immune infiltration [34]. Notably, aside from the ER localization, either ectopically expressed or endogenous RETSAT protein has obvious nuclear location [26, 35]. However, the exact nuclear functions of RETSAT are still misty.

In this study, we identified RETSAT as a novel replication fork binding protein. HIF-1α signaling promotes RETSAT expression under severe hypoxia. RETSAT associates with DDX39B on forks to unwind R-loops and promotes fork restarting, through which protects PDAC cells from fork damage and CHK1 initiated apoptosis. Targeting DDX39B using chemical CCT018159 sensitized PDAC cells and organoids to gemcitabine therapy.

## Materials and methods

### Cell lines and culture

The human pancreatic cancer cell lines PANC-1and BxPC-3 were purchased from Conservation Genetics CAS Kunming Cell Bank (Yunnan, China) and validated with short tandem repeat (STR) profiling. The human pancreatic duct epithelial cells HPDE6-C7 were obtained from China Center for Type Culture Collection (Hubei, China). Gemcitabine resistant PANC-1 subline (PANC-1/Gem-R) was purchased from China Center for Type Culture Collection (Hubei, China). PANC-1, BxPC-3 and HPDE6-C7 were cultured in RPMI-1640 containing 10% fetal bovine serum (Gibco, Cat. no.10099141C), 100 U/mL penicillin (Life Technologies, Cat. no. 15140122), and 100 mg/mL streptomycin (Life Technologies, Cat. no. 15140148). PANC-1/Gem-R cells were cultured in RPMI-1640 containing 10% fetal bovine serum (Gibco, Cat. no.10099141C), 100 U/mL penicillin (Life Technologies, Cat. no. 15140122), and 100 mg/mL streptomycin (Life Technologies, Cat. no. 15140148) and 2.5 μg/ml Gemcitabine (Selleck, Cat. no.s1714). All cells were regularly tested and confirmed for free of mycoplasma contamination using the LookOut Mycoplasma PCR detection (Sigma, Cat. No. MP0035).

### Reagents and antibodies

Thymidine was purchased from Sigma (Cat. no. T1895). Bromodeoxyuridine (BrdU) was purchased from Sigma (Cat. no. B5002). 5-ethynyl-2′-deoxyuridine (EdU) was purchased from Life Technologies (Cat. no. A10044). 5-Iodo-2-deoxyuridine (IdU) was purchased from Sigma (Cat. no. I7125). 5-chloro-2′-deoxyuridine (CIdU) was purchased from Sigma (Cat. no. C6891). Hydroxyurea (HU) was purchased from Sigma (Cat. no. H8627). Gemcitabine was purchased from Selleck (Cat. no. LY-188011). (T2AG3)-Cy3-labeled peptide nucleic acid telomeric probe was purchased from PANAGENE (Cat. no. F2001). Biotin-azide was purchased from Life Technology (Cat. no. B10184). Matrigel was purchased from BD Bioscience (Cat. no. 356234). D-Luciferin was purchased from BioVision (Cat. no. 7903). Green-fluorescent caspase 3/7 probe reagent was purchased from Invitrogen (Cat. no. R37111). SYBR™ Green was purchased from Life Technologies (Cat. no. A25778). FITC Annexin V apoptosis detection kit was purchased from BD Pharmingen (Cat. no. 556547). Low melting agarose was purchased from sigma (Cat. no. A9414). CHK1 antagonist PF47736 was purchased from MedChemExpress (Cat. no. HY-10032). ATR antagonist VE-821 was purchased from MedChemExpress (Cat. no. HY-14731). HIF1α antagonist PX-478 was purchased from MedChemExpress (Cat. no. HY-10231). HIF2α antagonist PT-2385 was purchased from MedChemExpress (Cat. no. HY-12867). Glycine was purchased from Sangon Biotech (Cat. no. A100167). Aprotinin was purchased from Sigma (Cat. no. A6103). Leupeptin was purchased from Sigma (Cat. no. L2884). Subcellular Protein Fractionation Kit was purchased from Thermo fisher (Cat. no. L78840). Streptavidin-agarose beads were purchased from Thermo fisher (Cat. no. 11205D). Organoid Dissociation Solution was purchased from BioGenous (Cat. no. E238001).

The following antibodies were obtained from the indicated suppliers: Rabbit anti-RETSAT (Invitrogen, Cat. no. PA5–65443, 1:500 for immunofluorescence and 1:200 for immunohistochemistry and 1:1000 for immunoblotting). Rat anti-BrdU (Abcam, Cat. no. 6326, 1:1000 for immunofluorescence). Rabbit anti-Phospho-Histone H2A.X(Ser139) (Cell Signaling Technology, Cat. no. 9718, 1:1000 for immunoblotting and 1:500 for immunofluorescence). Rabbit anti-DDX39B (Proteintech, Cat. no. 14798–1-AP, 1:500 for immunofluorescence and 1:1000 for immunoblotting). Mouse anti-DNA-RNA Hybrid [S9.6] (Kerafast, Cat. no. ENH001, 1:200 for immunofluorescence and 1:1000 for immunoblotting). Mouse anti-dsDNA (Santa Cruz, Cat. no.sc-58,749, 1:1000 for immunoblotting). Mouse anti-CHK1 (Cell Signaling Technology, Cat. no. 2360, 1:1000 for immunoblotting). Rabbit anti-Phospho-CHK1 (Ser345) (Cell Signaling Technology, Cat. no. 2348, 1:1000 for immunoblotting). Rabbit anti-Cleaved Caspase-3 (Cell Signaling Technology, Cat. no. 9664, 1:1000 for immunofluorescence). Mouse anti-Ki67 (Vector Laboratories, cat. no. VP-K452). Rabbit anti-ATR (Cell Signaling Technology, Cat. no. 2790, 1:1000 for immunoblotting). Rabbit anti- Phospho-ATR (Ser428) (Cell Signaling Technology, Cat. no. 2853, 1:1000 for immunoblotting). Mouse anti-H2B (Abcam, Cat. no. ab204463, 1:1000 for immunoblotting). Mouse anti-GAPDH (Cell Signaling Technology, Cat. no. 97166, 1:1000 for immunoblotting). Mouse anti-CK19 (Santa Cruz, Cat. no. sc-376,126, 1:200 for immunofluorescence). Rabbit anti-SMARCAL1 (Proteintech, Cat. no. 12513–1-AP, 1:1000 for immunoblotting). Rabbit anti-BLM (Affinit, Cat. no. DF13252, 1:1000 for immunoblotting). The secondary antibodies used for immunofluorescence were raised against rat (conjugated with Cyanine3, ThermoFisher, Cat. no. A-10522), rabbit (conjugated with Alexa 488, ThermoFisher, Cat. no. A-11008; conjugated with Alexa 555, ThermoFisher, Cat. no. A32732) or mouse (conjugated with Alexa 488, ThermoFisher, Cat. no. A11029; conjugated with Alexa 555, ThermoFisher, Cat. no. A31570). The secondary antibodies used for immunoblotting were raised against rabbit (conjugated with HRP, ThermoFisher, Cat. no. 31460) or mouse (conjugated with HRP, ThermoFisher, Cat. no. 31430).

### Constructs and lentiviral infection

The guide RNA (gRNA) sequences of *RETSAT* were obtained from GenScript’s gRNA Database (www.genscript.com/gRNA-database.html) and cloned into the lentiCRISPR v2 plasmid (Addgene plasmid # 52961) by Esp3I digestion (ThermoFisher, Cat. no. ER0451). The sequence of *RETSAT* gRNA was GGTGCTGGAACAACATACCA. pTomo-Luciferase-IRES-puro was constructed by replacing the RFP in pTomo-empty vector (Addgene plasmid #26291) with Luciferase by XbaI/BamHI digestion, the EGFP was replaced with puromycin resistant gene with BamHI/SalI digestion. pTomo-EF1a-Flag RNase H1 was constructed by inserting EF1α promoter in pTomo-empty by ClaI/XbaI digestion, and then inserted the 3 × Flag labeled RNase H1 fragment by XbaI/SalI digestion. The short hairpin RNAs shRNA targeting *DDX39B*, *BLM* and *SMARCAL1* were cloned into the pLKO.1-TRC cloning vector plasmid (Addgene plasmid # 10878) by AgeI/EcoRI digestion. Sequences of DDX39B shRNA 1# was: Forward: 5′-CCGGCCTCAACCTCAAACACATTAACTCGAGTTAATGTGTTTGAGGTTGAGGTTTTTG-3′; Reverse: 5′-AATTCAAAAACCTCAACCTCAAACACATTAACTCGAGTTAATGTGTTTGAGGTTGAGG-3′. Sequences of DDX39B shRNA 2# was: Forward: 5′- CCGGTGCCGCAAGTTCA TGCAAGATCTCGAGATCTTGCATGAACTTGCGGCATTTTTG-3′; Reverse: 5′- AATTCAAAAATGCCG CAAGTTCATGCAAGATCTCGAGATCTTGCATGAACTTGCGGCA-3′. Sequences of BLM shRNA 1# was: Forward: 5′- CCGGGACGCTAGACAGATAAGTTTACTCGAGTA AACTTATCTGTCTAGCGTCTTTTTG-3′; Reverse: 5′- AATTCAAAAAGACGCTAGACAGATAAGTTT ACTCGAGTAAACTTATCTGTCTAGCGTC-3′. Sequences of BLM shRNA 2# was: Forward: 5′- CCGGACCGAATCTCAATGTACATAGCTCGAGCTA TGTACATTGAGATTCGGTTTTTTG-3′; Reverse: 5′- AATTCAAAAAACCGAATCTCAATGTACATAGCTC GAGCTATGTACATTGAGATTCGGT-3′. Sequences of SMARCAL1 shRNA 1# was: Forward: 5′- CCGGGGAACTCATTGCAGTGTTTAACTCGAGTTAAACACTGCAATGAGTTCCTTTTTG-3′; Reverse: 5′- AATTCAAAAAGGAACTCATTGCAGTGTTTAACTCGAGTTAAACACTGCAATGAGTTCC-3′. Sequences of SMARCAL shRNA 2# was: Forward: 5′- CCGGTGCCCTCATTCTCTTCTTCAACCTCG AGGTTGAAGAAGAGAATGAGGGCATTTTTG − 3′; Reverse: 5′- AATTCAAAAATGCCCTCATTCTCTT CTTCAACCTCGAGGTTGAAGAAGAGAATGAGGGCA − 3′.

The lentiviral vectors were transfected into HEK293T cells along with the packaging plasmids pCMVΔ8.9 and pMD2.G at a ratio of 5:2.5:1 using Lipofectamine 3000 (Invitrogen, Cat. no. L3000015). Lentivirus was harvested 48 hours post transfection and filtered with 0.45 μm filter (Millipore, Cat. no. SLHV033RB). Pancreatic cancer cells were infected with lentiviruses and screened with 3 μg/mL puromycin 72 hours post infection.

### Immunofluorescence

Immunofluorescence was performed as described previously [36]. Specifically, for co-localization analysis of RETSAT with BrdU-labeled replication foci, cells were pulse labeled with 10 μM BrdU for 5 minutes. After 4% Paraformaldehyde fixation and treatment with 2 N HCl (Hydrochloric acid) at 4 °C overnight, cells were washed with PBS for three times to remove residual HCl, treated with 0.3% Triton X-100 for 15 minutes, and blocked by 10% goat serum for 1 hour at room temperature. Then cells were incubated overnight at 4 °C with primary antibodies, and then labeled by fluorescent second antibodies for 1 hour at room temperature. Nucleus was stained by DAPI. Images were captured using confocal microscope system (Olympus, FV1000).

### Immunoblotting

Cells were lysed in RIPA buffer containing protease inhibitor cocktail (ThermoFisher, Cat. no. 87786) and centrifuged to remove the debris. Concentration of supernatant protein was quantified with BCA method (Beyotime; Cat. no. P0009). Standard SDS-PAGE gel electrophoresis was performed, followed by blocking with 5% skimmed milk and immunoblotting with primary antibodies at 4 °C overnight. Specific signals were detected with horseradish peroxidase-conjugated secondary antibodies and chemiluminescent horseradish peroxidase substrate reagents (Millipore, Cat. no. WbKLS0500). Images were captured using automatic chemiluminescence imaging analysis system (Tanon, 5200).

### Dot blotting

Cells were trypsinized and washed twice with ice-cold PBS, and lysed by cell lysis buffer (0.5% NP-40, 80 mM KCl, 5 mM PIPES) for 10 minutes. The nuclear was obtained through centrifuge at 500 x g for 5 minutes, and lysed with nuclear lysis buffer (1% SDS, 25 mM Tris-HCl pH 8.0, 5 mM EDTA) for 10 minutes. Lysis was added into 3 μL 20 mg/mL proteinase K and incubate for 3–5 hours at 55 °C. Extraction was performed twice using phenol:chloroform:isoamyl alcohol (25:24:1, pH 8.0) and chloroform, followed by 3 M sodium acetate (pH 5.2), glycogen and ice-cold 100% ethanol. After spinning down at 12,000 x g for 30 minutes at 4 °C and washing with 1 mL 70% ethanol, the pellet was resuspended into elution buffer (10 mM Tris-Cl, pH 8.5). Genomic DNA was diluted in 50 μL TE buffer and spotted onto Hybond N+ membrane (GE Healthcare) using a Bio-Dot Apparatus (Bio-Rad, Cat. no.1706545,). The membrane was blocked with 5% skim milk at room temperature after ultraviolet (UV) (0.24 J) cross-linking for 1 hour. The membrane was incubated with S9.6 antibody or dsDNA antibody overnight at 4 °C, followed by procedures as same as immunoblotting described above.

### Immunohistochemistry

PDAC microarray was purchased from Shanghai Outdo Biotech (HPan-Ade180Sur-01). The clinical pancreatic tumor tissues were obtained from The Second Affiliated Hospital of Kunming Medical University. The protocol was approved by the Human Resource Use Committee of The Second Affiliated Hospital of Kunming Medical University. The immunohistochemistry (IHC) was performed as described [37]. Briefly, sections were deparaffinized and rehydrated, and antigen retrieval was performed in citric acid solution (pH 6.0) for 5 minutes at 125 °C in an autoclave. Endogenous peroxidase activity was quenched by incubation in 3% hydrogen peroxide for 15 minutes, followed by blocking in 10% goat serum for 1 hour at room temperature, incubation overnight at 4 °C with primary antibodies, and HRP-DAB staining (Beyotime, cat. no. P0202) or fluorescent secondary antibody. The slides were mounted with Aqua-Poly/Mount (cat. no. 18606; Polysciences, Warminster, PA, USA). Images were captured using Three-dimensional ultra-depth-of-field microscope VHX-6000 and Olympus optical microscope BX43.

### Clone formation assay

Colony formation assay in soft agar was performed as described previously [38]. Basal agarose layer (0.8%) was prepared of 1 mL for one 6-well plate by diluting stock agar solution with growth medium and cooled at 4 °C for ~ 5 minutes. The upper agarose layer (0.48%) was mixed well with 10^4^ cells and immediately dropped onto solidified basal layer, then cooled at 4 °C for 5 minutes. One milliliter of growth medium was added. Cells were incubated at 37 °C and 5% CO_2_ for 20 days. Cultural medium was refreshed every 4–7 days. The clones were staining by 0.005% crystal violet and counted using the dissecting microscope.

### Cell apoptosis assay

For monolayer cultured cells, apoptosis analysis was performed by flow cytometry using FITC Annexin V apoptosis detection kit I (BD Pharmingen, Cat. no. 556547) according to manufacturer’s guidance. For 3D spheroids, apoptosis analysis was performed by green-fluorescent caspase 3/7 probe reagent and flow cytometry using FITC Annexin V apoptosis detection kit I (BD Pharmingen, Cat. no. 556547) after dissociated with Organoid Dissociation Solution (E238001), green-fluorescent caspase 3/7 probe reagent was added into medium and incubated for 30–60 minutes. The green fluorescence was observed with fluorescence microscope, the density of fluorescence was quantified with Image J software.

### Real-time RT-PCR

Total RNA was extracted using Trizol reagent (sigma, Cat. no.T9424). Reverse transcription was carried out using the RevertAid First Strand cDNA Synthesis kit (Thermo Fisher Scientific, Cat.no.K1621). Quantitative real-time PCR was performed using SYBR Select Master Mix kit (Life Technologies, Cat. no. A25778). The primers used for *RETSAT* were: forward 5′-ATTGCCTTCCACACCATC-3′, reverse 5′-TTGAACAGTCCTGCGTTG-3′.

### Neutral comet assay

The neutral comet assay was performed as described [39]. Briefly, 2 × 10^3^ cells in 10 μL PBS were added into 70 μL 1% low-melting agarose at 37 °C, pipetted and evenly spread onto slide pre-coated with 0.8% agarose. The slides were incubated at 4 °C in the dark for 10 minutes, and then transferred into prechilled lysis solution (2.5 M NaCl, 100 mM EDTA, 10 mM Tris-base, 1% sodium lauryl sarcosinate, 1% Triton X-100, pH 9.5) for 60 minutes at 4 °C. The slides were then transferred to prechilled neutral electrophoresis solution (300 mM sodium acetate, 100 mM Tris, pH = 8.3) and subjected to electrophoresis at 15 V/cm, 80 mA for 30 minutes, followed by washing with distilled water and immersed in ice cold 100% ethanol at room temperature for 5 minutes and air dried. DNA was stained with DAPI for 5 minutes. Comets were analyzed using Comet Assay Software Project (CASP) (Andor Technology). A total of 150 cells from different random areas were counted per slide. Each experiment was repeated at least twice independently.

### DNA fiber assay

DNA fiber assay was performed as described [40]. Specifically, replicating DNA was first labeled with 25 μM 5-iodo-2′-deoxyuridinefor for 20 minutes with or without HU treatment. Cells were then subjected to the second labeling with 250 μM 5-chloro-2′-deoxyuridine. After labeling, 2.5 μL of the cell suspension (∼2500 cells) were spotted onto one end of the glass slide, followed by addition of 7.5 μL of lysis buffer (50 mM EDTA, 0.5% SDS, 200 mM Tris-HCl, pH 7.5). After incubation for 8 minutes at room temperature, the slides were tilted to 15° to allow the DNA fibers to spread down along the slide. DNA fibers were treated with 2.5 M hydrochloric acid and incubated with rat anti-BrdU monoclonal antibody that recognizes CIdU, but not IdU at 4 °C overnight, followed by an AlexaFluor cy3-conjugated goat anti-rat secondary antibody for 1 hour at room temperature. The mouse anti-IdU monoclonal antibody that recognizes IdU but not CIdU (4 °C overnight) and AlexaFluor 488-conjugated goat anti-mouse secondary antibody (1 hour at room temperature) were used to detect IdU. DNA fibers were analyzed on a Leica DM6000B microscope equipped with a CoolSNAP HQ CCD camera (Roper Scientifics). The lengths of CIdU (AF cy3, red) and IdU (AF 488, green) labeled patches were measured using the Image J software, and μm values were converted into kb using the formula 1 μm = 2.59 kb. Two hundred fibers from different random areas were analyzed for assessment of fork dynamics.

### Isolate proteins on nascent DNA (iPOND)

iPOND was performed as described [41, 42]. Briefly, pancreatic cancer cells were cultured under normal conditions with or without gemcitabine. Cells were synchronized in S phase by twice treatment of thymidine. For the first time of treatment, cells were treated with 2 mM thymidine for 18 hours, followed by release into thymidine-free medium for 10 hours. Then the second treatment was performed with 2 mM thymidine for 18 hours, and released for 6 hours. Cells were incubated with 10 mM EdU for 10 minutes. After EdU labeling, cells were treated with or without gemcitabine for 4 hours. Cells were then fixed in 1% formaldehyde, followed by quenching with 0.125 M glycine (Sangon Biotech, A100167). Cells were then collected and washed three times in ice-cold PBS, and permeabilized in ice-cold 0.25% Triton X-100/PBS for 30 minutes. Before click reaction, samples were washed once in 0.5% BSA/PBS and once in ice-cold PBS.

For click reaction, cells were incubated in click reaction buffer for 1 hour at room temperature containing 10 μM Biotin-azide. The “no-click” sample (negative control) used DMSO instead of Biotin-azide. Following the Click reaction, cells were washed once in 0.5% BSA/PBS and once in ice-cold PBS, and resuspended in lysis buffer (50 mM Tris-HCl, pH 8.0, 1% SDS) containing 1 μg/mL aprotinin (Sigma, A6103) and 1 μg/mL leupeptin (Sigma, L2884) and sonicated using a BioruptorTM UCD-200 for 60 cycles (30s pulse/ 30s pause). Samples were centrifuged at 16100×g at 4 °C for 10 minutes and the supernatant was collected. The supernatant was filtered through a 90-μm nylon mesh and diluted 1:1 (*V/V*) with ice cold PBS containing 1 μg/mL aprotinin and 1 μg/mL leupeptin. The input samples were collected. Streptavidin-agarose beads (Thermo fisher, 11205D) were washed three times in lysis buffer containing aprotinin and leupeptin. Two hundred microliter bead slurry was used for 1 × 10^8^ cells. The streptavidin-agarose beads were added to the samples, which were then incubated at 4 °C for 16 hours in dark. Following binding, the beads were washed with ice-cold lysis buffer, followed by one wash with 1 M NaCl and two washes with ice-cold lysis buffer. To elute proteins bound to nascent DNA, the 2× SDS Laemmli sample buffer (2× SB) mix (0.4 g SDS, 2 mL 100% Glycerol, 1.25 mL 1 M Tris, pH 6.8 and 0.01 g Bromophenol blue in 8 mL H_2_O) was added to packed beads (1:1; *V/V*). Samples were incubated at 95 °C for 25 minutes, followed by immunoblotting or mass spectrometry detection.

### Mass spectrometry assay

The purified proteins were separated by SDS-PAGE and visualized by silver staining. The gel was then cut into small pieces. Disulfide bonds were reduced, thiols were alkylated, and proteins were digested according to the in-gel trypsin digestion protocol [43]. The extracted peptides were dried, resuspended in 0.1% trifluoroacetic acid, desalted with C18 ZipTips, dried again, and dissolved in 0.1% formic acid.

An Orbitrap Elite hybrid mass spectrometer (MS) with an electrospray ionization inlet (Thermo Fisher) was used to analyze the peptide samples using a previously described method [44]. Briefly, samples were separated on a C18 analytical column through a nanoscale HPLC with solution A of 0.1% formic acid and solvent B of 80% acetonitrile and 0.1% formic acid. The HPLC gradient was 6 to 44% solvent B for 90 minutes. The automatic data acquisition in positive ion mode in MS was used to collect the 15 strongest ions in each precursor MS scan. Each precursor ion was analyzed twice in 60 seconds. The resolution for the precursor ion was set to 120,000 at 200 m/z and the isolation window of the selected precursor ion for MS/MS analysis was set to 2 m/z.

The MS/MS raw files were searched with Proteome Discoverer (version 2.1, Thermo Fisher Scientific) against the Human UniProt database (www.uniprot.org) with concatenated reverse protein sequence and common contaminants. The parameters used to identify tryptic peptides for the protein identification were a 10 ppm precursor-ion mass tolerance, 0.6 Da production mass tolerance. Enzyme specificity was set to trypsin and a maximum of 2 missed cleavages per peptide were allowed. The cysteine carbamidomethylation was set as fixed modification and methionine oxidation and N-terminal acetylation as variable modifications. The 1% FDR at both peptide and protein levels was applied for the analysis. Relative protein quantification was based on the label-free quantification included in the Proteome Discoverer software package. The Abundance of the protein was obtained from each sample.

### Co-immunoprecipitation

Co-immunoprecipitation (Co-IP) was performed as described previously [36]. Specifically, PANC-1 cells were harvested and washed twice with ice-cold PBS and lysed with 1× RIPA lysis buffer (Beyotime, P0013D) containing complete EDTA-free (Roche) inhibitors. Immunoprecipitation with RETSAT antibody performed on and with Lysates were digested by 10 units/mL DNase I (New England Biolabs, M0303), and incubated with anti-RETSAT primary antibody overnight. Isotype IgG were used as negative controls. Immunoprecipitation was carried out using protein A/G Agarose Resin (Yeasen, 36403ES08) according to the manufacturers’ protocol. After pulling down and wash, proteins were fractionated by SDS-PAGE gel for immunoblotting.

### 3D spheroid culture

3D culture of pancreatic cancer cells was performed as previous described [45]. Briefly, Matrigel was diluted with serum-free culture medium to a final concentration of 7 mg/mL. One hundred microliter diluted Matrigel was added into each well of 96-well plate and incubated for 60 minutes in 37 °C for solidification. Pancreatic cancer cells were seeded onto Matrigel at a density of 5000 cells/well. Gemcitabine was added the next day post cell seeding and maintained for a total of 7 days, with a midweek change of fresh medium.

### PDAC organoids culture

Primary human PDAC organoids were established from two PDAC surgical biopsies in The Second Affiliated Hospital of Kunming Medical University as previously described [46–48]. Briefly, human PDAC tissues were rinsed with DPBS twice and minced into small fragments of 1–3 mm^3^, followed by digestion with 10 mL of tumor tissue digestion solution (BioGenous, K601003) in a 15 mL conical tube at 37 °C for variable incubation times ranging from 30 min to 90 min. Cells were filtered using a 100 μm cell strainer and centrifuged at 250×g for 3 min at 4 °C. Cells were resuspended with AdDMEM/F12 (Invitrogen,12,634–010) and Growth Factor reduced (GFR) Matrigel (Corning, 356,231). Thirty microliter matrigel containing approximately 10,000 cells was loaded onto the bottom of 24-well plates and incubated at 37 °C and 5% (*vol/vol*) CO_2_ for 25 min for solidification. The culture medium was composed of AdDMEM/F12 (basal medium), 1 M HEPES, 1x GlutaMax (Invitrogen, 35,050–061), 1% penicillin/streptomycin (Invitrogen, 15,140,122), 1x B27 (Invitrogen, 17,504,044), 1 mM N-acetyl-L-cysteine (Sigma-Aldrich, 9165), 100 ng/mL Wnt-3a (R&D Systems, 5036-WN-010), 100 ng/mL R-Spondin 1 (Peprotech, 120–38), 100 ng/mL Noggin (Invitrogen, 120-10C), 50 ng/ml epidermal growth factor (Peprotech, AF-100-15), 100 ng/mL fibroblast growth factor (Peprotech, C100–26), 10 mM Nicotinamide (Sigma, N0636), 10 μM Y-27263 (Sigma, Y0503) and 0.5 μM A83–01 (R&D Systems, 2939/10).

### Cell Derived Xenograft (CDX) model

Animal care and experimental protocols were approved by the Institutional Animal Care and Use Committee of Kunming Institute of Zoology, Chinese Academy of Sciences. Five to six-week-old B-NDG (NOD-Prkdcscid IL2rgtm1/Bcgen) mice were purchased from Jiangsu Biocytogen Co., Ltd. (Nantong, China) and kept under specific pathogen-free environment. PANC-1 cells were infected with lentivirus expressing Luciferase and selected with 3 μg/mL puromycin for 7 days. 1 × 10^6^ cancer cells in 100 μL PBS containing 30% Matrigel were injected into each B-NDG mouse subcutaneously. To monitor tumor growth by bioluminescent imaging in vivo weekly, the mice were intraperitoneally injected with 150 mg/kg D-Luciferin and imaged using IVIS system followed by analyzed with Living Image software (Caliper Life Science, IVIS Lumina Xr, USA). Mice were treated with 100 μL of vehicle (saline) or gemcitabine at dose of 50 mg/kg by intraperitoneal injection weekly. The mice were sacrificed before tumor volume reached to approximately 2000 mm^3^.

### Statistical analysis

Statistical analysis was performed using GraphPad Prism 9 software (GraphPad Inc., San Diego, CA, USA). Quantitative data are represented as mean ± s.e.m. unless otherwise stated. Comparisons between two groups were analyzed by two tailed Student’s *t*-test for statistical significance. One-way analysis of variances was applied for multiple comparisons. Experiments were repeated three times unless otherwise stated. No samples or animals were excluded from any analyses and all replicates were authentic biological replicates. Animals were randomly assigned to treatment of gemcitabine. Blind analysis was not performed in this study. *P* < 0.05 was considered as significant.

## Results

### High expression of RETSAT correlated to poor survival in PDAC patients

**Fig. 1:**
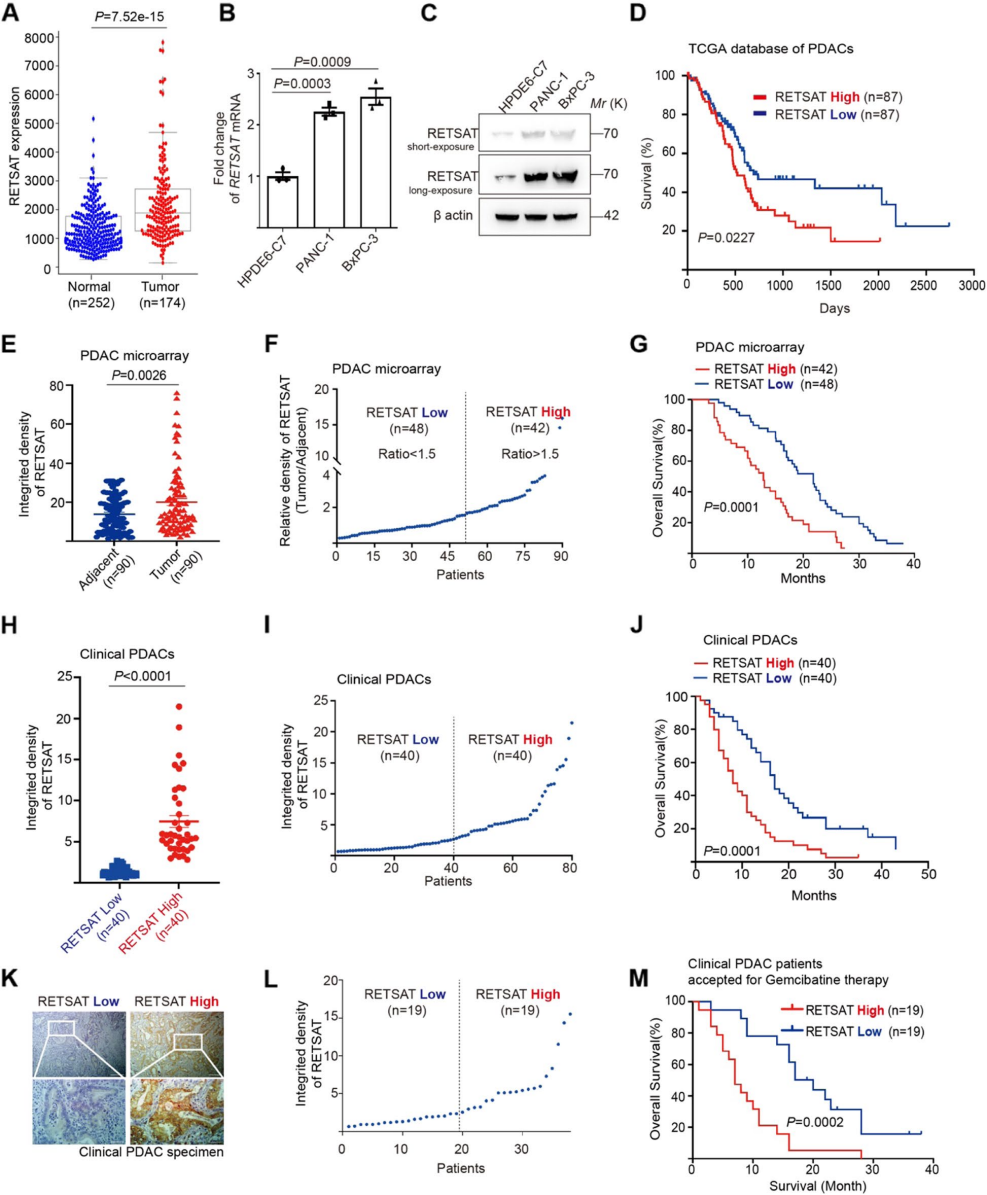
High expression of RETSAT correlated to poor survival in PDAC patients. **A** The expression of *RETSAT* in PDAC tumor tissues (*n* = 174) from The Cancer Genome Atlas database compare to normal tissues from The Cancer Genome Atlas database and GTEx database (*n* = 252). **B,C** qPCR (**B**) and immunoblotting analysis (**C**) of RETSAT in human pancreatic duct epithelial (HPDE) cell line HPDE6-C7, pancreatic cancer cell lines PANC-1 and BxPC-3, the exposure time was 0.2 s (short exposure) and 1 s (long exposure) respectively in (**C**). **D** Kaplan-Meier curve for overall survival of PDAC patients (*n* = 174) with low vs high expression of RETSAT. The data was downloaded from TCGA dataset and re-analyzed. **E** Quantification of IHC integrated density of RETSAT in PDAC tissue microarray using image J software. Ninety adjacent tissues and corresponding tumor tissues were calculated. **F** Classification of PDAC microarray tissues into RETSAT high (*n* = 42) and low (*n* = 48) subgroups based on the ratio of RETSAT integrated density in tumor tissues versus own adjacent tissues. Tissues with ratio greater than 1.5 were defined as RETSAT-high, while lower than 1.5 were defined as RETSAT-low. **G** Overall survival time (months) of RETSAT-high and RETSAT-low subgroups based on microarray information. The figure shows the Kaplan–Meier survival curves. **H, I** Quantification of IHC integrated density of RETSAT in PDAC clinical tissues using image J software (**H**), 80 tumor tissues were calculated and divided into RETSAT high (*n* = 40) or low (*n* = 40) subgroups (**I**) based on IHC integrated density of RETSAT. **J** Kaplan–Meier curve for overall survival of RETSAT-high and RETSAT-low subgroups based on clinical information. **K** Representative images of immumohistochemistry staining of RETSAT in clinical PDAC tissues. **L** 38 PDAC tissues collected from clinical surgeries ahead of gemcitabine based treatment were classified into RETSAT high (*n* = 19) or low (*n* = 19) subgroups based on IHC integrated density of RETSAT. **M** Kaplan–Meier curve for overall survival of PDAC patients after surgery and followed by gemcitabine based therapy. Scale bar = 200 μm in (**K**), *n* = 3 independent experiments unless otherwise stated. All data are presented as mean ± SEM. *P* values were calculated using a two-tailed student’s *t* test

To study the roles of *RETSAT* in pancreatic cancers, we first downloaded the bulk transcriptional database from TCGA (The Cancer Genome Atlas). Compared with normal pancreatic tissues (*n* = 252 cases), the *RETSAT* mRNA levels were dramatically high in tumor tissues (*n* = 174 cases) (*P* = 7.52 × 10^− 15^, Fig. 1A). We further examined its expression in non-transformed human pancreatic duct epithelial (HPDE) cell line H6c7 and transformed PDAC cell lines BxPC-3 and PANC-1. Compared to H6c7, RETSAT was overexpressed in BxPC-3 and PANC-1 at both mRNA transcription and protein levels (Fig. 1B-C). Notably, PANC-1 cells have *KRAS*^*G12D*^ and *TP53*^*R273H*^ mutation genetically. BxPC-3 cells have wide type *KRAS* and *TP53*^*Y220C*^ mutation [49]. Furthermore, we compared the transcription of RETSAT in *KRAS* mutant PDAC tumors (*n* = 86) with *KRAS* wide type counterparts (*n* = 10) in TCGA dataset (Supplementary Fig. 1A), showing that there was no significant difference (*P* = 0.68). These results indicate that *RETSAT* is highly expressed in transformed PDAC cells regardless of *KRAS* genetic status.

We next focused on 174 PDAC cases used in Fig. 1A. These cases were ranked based on the FPKM (Fragments Per Kilobase Million) of *RETSAT*, and defined as *RETSAT* high (50% of total, 87 cases) or *RETSAT* low (50% of total, 87 cases) subgroups. We found that the *RETSAT* level conversely related to PDAC overall survival, with high RETSAT corresponding to poor survival compared with low subgroup (*P* = 0.027, Fig. 1D). For validation, we performed immunohistochemistry (IHC) of RETSAT in commercial PDAC microarray, which included 90 PDAC specimens. Compared with own adjacent, the integrated density of RETSAT was drastically higher in tumor regions (*P* = 0.0026, Fig. 1E). Based on the ratio of RETSAT integrated density in tumor area versus own adjacent. Forty-eight samples with ratios less than 1.5 were defined as low, while 42 samples with ratios greater than 1.5 were defined as high (Fig. 1F). Consistent to analysis from TCGA database, RETSAT-high subgroup in PDAC microarray showed significantly poor survival compared with low subgroup (*P* = 0.0001, Fig. 1G). We also collected 80 clinical PDAC specimens for confirmation. As such, the expression of RETSAT in 40 cases was quite higher than the rest ones (*P* < 0.0001, Fig. 1H). Through separating them into low (40 cases) and high (40 cases) subgroups (Fig. 1I), we got the similar tendency between RETSAT expression and survival (*P* = 0.0001, Fig. 1J).

Among these clinical specimens, 38 cases came from PDAC patients who accepted for PDAC clinical surgery, followed by gemcitabine based chemotherapy. These specimens were originally from clinical operation without any chemical treatments ahead of surgeries. After surgeries, all 38 patients accepted for gemcitabine, gemcitabine plus Albumin Paclitaxel, gemcitabine plus Cisplatin based therapy. We examined the expression of RETSAT in each specimen by means of IHC. Meanwhile, the survival time of each patient after surgery was confirmed through telephone communication with patients in person or their immediate families. The specimens were ranked based on RETSAT level (Fig. 1K-L). After integrated analysis, we found that patients with low level of RETSAT were benefitted more from gemcitabine therapy (*P* = 0.0002, Fig. 1M). Together, these findings support the notion that high RETSAT is related to poor survival in the context of PDAC.

### *RETSAT* deletion sensitized PDAC cells to gemcitabine induced apoptosis

**Fig. 2:**
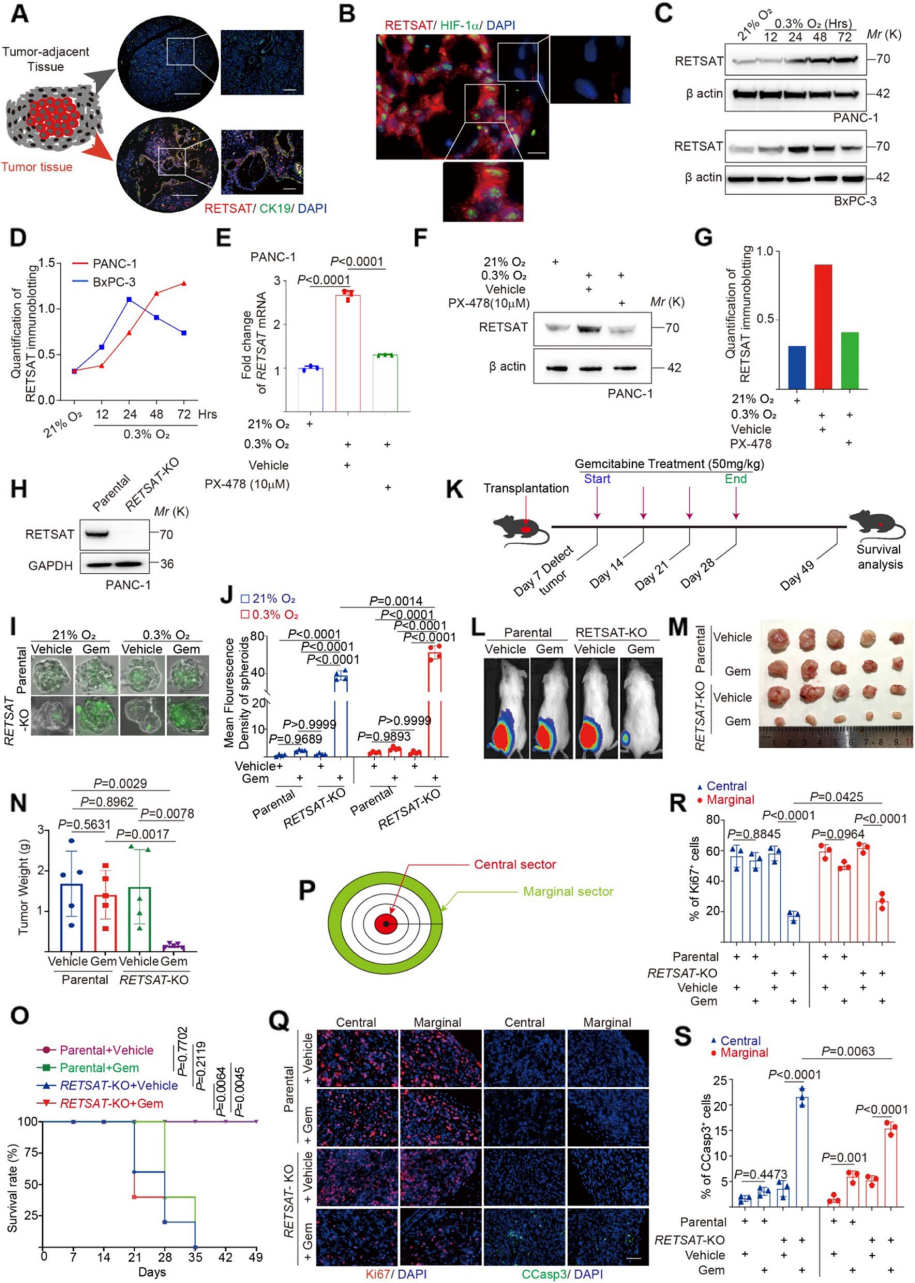
RETSAT deletion sensitized PDAC cells to gemcitabine induced apoptosis. **A** Representative images of fluorescent Co-immunohistochemistry staining of RETSAT(red) and ductal cell marker CK19 (green) in PDAC tissue microarray. **B** Co-immunohistofluorescence of RETSAT (red) and HIF-1α (green) in PDAC tissue. **C, D** Immunoblotting (**C**) and quantification (**D**) of RETSAT in PDAC cell lines PANC-1 and BxPC-3. Cells were cultured under normoxia (21% O_2_) or hypoxia (0.3% O_2_) for 12, 24, 48, 72 hours respectively. β actin was used as a loading control. **E** qPCR analysis of *RETSAT* transcription under indicated treatments. HIF-1α antagonist PX-478(10 μM) was used. **F, G** Immunoblotting (**F**) and quantification (**G**) of RETSAT in PANC-1 cells under indicated treatments. **H** Immunoblotting confirmation of RETSAT deletion in luciferase positive PANC-1 cells. **I, J** Representative images (**I**) and quantification (**J**) of apoptosis using green-fluorescent caspase 3/7 probe labeling in 3D cultured PANC-1 spheroids with or without RETSAT under indicated treatments. **K** Experimental setup and treatment schedule of CDX assay. Tumor sizes were detected by IVIS system at day 7 post transplantation, and starting Gemcitabine treatment at dose of 50 mg/kg weekly and ending at day 28, the mice were monitored until day 49. **L** Representative images of bioluminescence signals in mice bearing with PANC-1 cell derived xenografts. **M, N** Image of cell derived xenografts (**M**) and weight quantification (**N**) from PANC-1 parental and *RETSAT* knockout cells after gemcitabine therapy. **O** Survival curve of mice bearing with PANC-1 cell derived xenografts under indicated therapy. The figure shows the Kaplan–Meier survival curves (*n* = 5 biologically independent mice per group). **P** Definition of tumor central and marginal sectors. **Q-S** Representative images (**Q**) and quantification of Ki67 (**R**) and cleaved caspase 3 (**S**) immunohistofluorescence in CDXs central and marginal sectors, respectively. Scale bar = 200 μm in (**A**), 50 μm in (**B**), 100 μm in (**I**) and (**Q**). *n* = 3 independent experiments unless otherwise stated. All data are presented as mean ± SEM. *P* values were calculated using a two-tailed student’s *t* test

We examined the location of RETSAT in PDAC specimens. Notably, dramatic RETSAT was observed in PDAC ductal regions (Fig. 2A), where has been defined as a major hypoxic area of PDAC [50]. To confirm this, we co-stained RETSAT with hypoxia marker HIF-1α in PDAC specimens. As shown in Fig. 2B, HIF-1α positive sectors had quite high level of RETSAT (right zoomed region, Fig. 2B), while HIF-1α negative sectors showed almost no RETSAT expression (lower zoomed region, Fig. 2B). This reminded us to investigate whether *RETSAT* is under control of HIF-1α signaling. To this goal, we cultured PANC-1 and BxPC-3 cells in 0.3% oxygen tension in order to mimic PDAC pathological hypoxia [2]. Cells were collected at consecutive time points for immunoblotting and qRT-PCR. We found that severe hypoxia could increase RETSAT level dramatically (Fig. 2C-E). PX-478 is a selective inhibitor of HIF-1α [51]. We treated PANC-1 cells with vehicle or 10uM PX-478 for 24 hours in 0.3% oxygen tension. Both qRT-PCR and immunoblotting results revealed that PX-478 significantly inhibited RETSAT level (Fig. 2E-G). This indicates that the upstream HIF-1α signaling promotes RETSAT expression in PDAC cells under severe hypoxia.

To investigate the roles of RETSAT in PDAC cell fate determination, we derived *RETSAT* knockout (*RETSAT*-KO) PANC-1 cells from luciferase positive parental (Fig. 2H). Monolayer (2-D) or spheroid (3-D) cultured cells were treated with or without 10 μM gemcitabine under 21% or 0.3% O_2_ oxygen tension for 72 hours, and collected for flow cytometry analysis. Compared to parental counterpart, *RETSAT*-KO cells proliferated more slowly under gemcitabine treatment, as evaluated by anti-Ki67 antibody immunostaining combined with flow cytometry analysis (*P* < 0.0001 *RETSAT*-KO versus parental under Gem in Supplementary Fig. 1B-C). Moreover, *RETSAT*-KO cells were more sensitive to apoptosis under gemcitabine and severe hypoxia conditions (*P* = 0.0004 *RETSAT*-KO versus parental under Gem and 0.3% O_2_ in Fig. 2I-J, *P* = 0.0014 *RETSAT*-KO versus parental under Gem and 0.3% O_2_ in Supplementary Fig. 1D-G). We validated this result in cell-derived xenografts (CDX) mice model (Fig. 2K). PANC-1 parental and *RETSAT*-KO cells were injected into NOD-SCID immunodeficient mice to form xenografts in parallel. One week post cell injection, mice bearing xenografts were separated into groups randomly for gemcitabine treatment. *RETSAT* knockout has no influence on CDX formation (*P* = 0.1781 parental + vehicle versus *RETSAT*-KO + vehicle, Supplementary Fig. 1H-I). After 3 weeks treatment of gemcitabine (50 mg/kg, once per week), gemcitabine suppressed the growth of *RETSAT*-KO tumors with decreased luciferase strength (*P* = 0.0023 parental + Gem versus *RETSAT*-KO + Gem, Supplementary Fig. 1 J-M), lower tumor size and weight (*P* = 0.0017 parental + Gem versus *RETSAT*-KO + Gem, Fig. 2L-N), and prolonged survival time (*P* = 0.0045 *RETSAT*-KO versus parental after gemcitabine treatment, Fig. 2O).

Xenografts were then fixed for immunohistochemistry (IHC) analysis. Central regions are defined as extreme hypoxia sectors of solid tumors where impede efficient PDAC chemotherapy [52]. Based on the diameter of each tumor, we defined the inner 10% area as central sector, and the outer 10% area as marginal sector (Fig. 2P). We used anti-Ki67 antibody to detect proliferating cells, and anti-cleaved caspase 3 (CCasp3) antibody to detect apoptosis. Regarding cell proliferation, there was no difference in parental central sectors (*P* = 0.8845 Fig. 2Q-R), and a bit decrease in parental marginal sectors but without statistical significance (*P* = 0.0964 Fig. 2Q-R), while in *RETSAT* knockout tissues, RETSAT deletion caused dramatic decrease of cell proliferation in both central and marginal sectors of CDXs (*P* < 0.0001 Fig. 2Q-R), and central sectors contained less proliferating cells compared to their own marginal counterparts (*P* = 0.0425 Fig. 2Q-R). Notably, In terms of apoptosis, *RETSAT* knockout CDXs contained more apoptotic cells in both central and marginal sectors of CDXs (*P* < 0.0001), with more apoptotic cells in central regions (*P* = 0.0063 Fig. 2Q-S). Together, we concluded that RETSAT knockout sensitized PDAC cells to apoptosis in gemcitabine treatment.

### RETSAT promotes fork restarting under replication stress

**Fig. 3:**
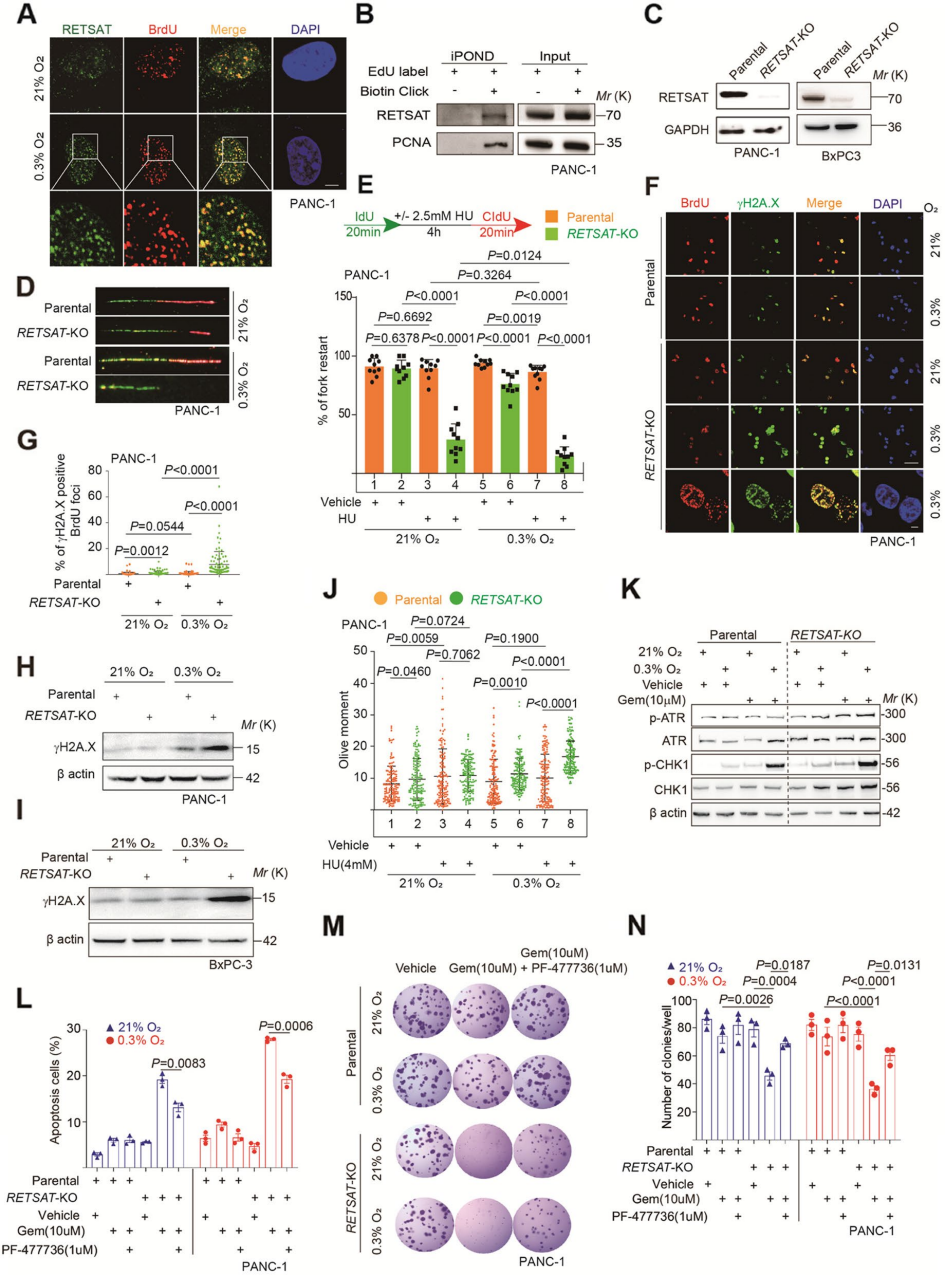
RETSAT promotes fork restarting under replication stress. **A** Co-immunostaining of RETSAT (green) and BrdU pulse labeled replication foci (red) in PANC-1 cells cultured under normoxia (21% O_2_) or hypoxia (0.3% O_2_). **B** iPOND assay to validate location of RETSAT on replication forks. PCNA was included as a positive control. **C** Immunoblotting of RETSAT in PANC-1 and BxPC-3 infected with or without RETSAT CRISPR gRNA lentivirus. β actin was used as a loading control. **D, E** Representative images (**D**) and quantification (**E**) of fork restarting in parental or *RETSAT*-KO PANC-1 cells under indicated treatments. 2.5 mM Hydroxyurea (HU) was used to induce replication stress. At least 200 single forks were calculated in each sample. **F, G** Representative images (**F**) and quantification (**G**) of replication fork damage in PANC-1 parental and *RETSAT*-KO cells under indicated treatments. Pulse labeled BrdU foci was indicating DNA replication sites. γH2A.X was used to indicate DNA damage. **H, I** Immunoblotting of γH2A.X in parental and *RETSAT*-KO PANC-1 (**H**) and BxPC-3 (**I**) under indicated treatments. β actin was used as a loading control. **J** Quantification of neutral comet assay in parental and *RETSAT*-KO PANC-1 cells under indicated treatments. At least 150 single comets were calculated in each sample. **K** Immunoblotting of ATR, p-ATR (Ser428), CHK1, p-CHK1 (Ser345) andβ actin in parental and *RETSAT*-KO PANC-1 cells under indicated treatments. **L** Flow cytometry based Annexin V apoptosis quantification in parental and *RETSAT*-KO PANC-1 cells under indicated treatments. 1 μM PF-477736 was used to inhibit CHK1 activity. **M, N** Representative images (**M**) and quantification (**N**) of clone formation assay of parental or *RETSAT*-KO PANC-1 cells under indicated treatments. Scale bar = 10 μm in (**A**), 50 μm in upper four panels and 10 μm in lowest panel in (**F**). *n* = 3 independent experiments unless otherwise stated. All data are presented as mean ± SEM. *P* values were calculated using a two-tailed student’s *t* test

Our in vitro and in vivo results consistently revealed that *RETSAT* knockout sensitized PDAC cells to gemcitabine induced apoptosis under severe hypoxia. We next sought to explore the mechanism. To this goal, we performed immunofluorescence in PDAC cells first to check the subcellular localization of RETSAT. In PANC-1 cells, RETSAT localized in both cytoplasm and nuclear (Supplementary Fig. 2A), consistent to published study [26]. After 0.2% Triton X-100/PBS pre-wash before paraformaldehyde fixation, cytoplasmic and dissociative RETSAT proteins were released. Then we observed that the remaining RETSAT showed as minor foci in nuclear morphologically (Fig. 3A). We first hypothesized these RETSAT foci might be correlated with telomere, since telomere always exhibits as small foci in the cellular nuclei when performing telomeric fluorescence in situ hybridization (T-FISH) assay [53]. However, we observed negative co-localization between telomeres and RETSAT foci in anti-RETSAT immunofluorescence combined with T-FISH assay (Supplementary Fig. 2B). Next, we tested the correlation of RETSAT with another form of minor foci named DNA replication foci when performing BrdU pulse labeling [40]. Obviously, RETSAT showed almost 100% co-localization with replication foci in PANC-1 (Fig. 3A) and BxPC-3 (Supplementary Fig. 2C) cell lines. Neither hydroxyurea (HU) induced replication stress nor severe hypoxia (0.3% oxygen tension) changed this co-localization (Supplementary Fig. 2C-E), indicating a constitutive manner of RETSAT on DNA replication sites.

iPOND (isolate proteins on nascent DNA) assay allows to examine proteins associated with replicating and newly synthesized DNA in mammalian cells, based on EdU pulse labeling of nascent DNA and covalent crosslink to Biotin through Cu^II^ catalyzed click chemistry reaction. Biotin linked nascent DNA fragments can be enriched by streptavidin-based affinity purification [41]. To confirm RETSAT is a fork associated protein, we performed iPOND assay in PANC-1 cells. PCNA was included as a positive control. After immunoblotting of iPOND samples, we clearly observed that RETSAT was pulled down from nascent DNA (Fig. 3B). Together, we concluded that RETSAT is a fork binding protein. Notably, under hydroxyurea (HU) or gemcitabine induced replicative stress [54], the protein level of RETSAT did not increase in PANC-1 cells (Supplementary Fig. 2F-G).

We investigated the correlation between gemcitabine induced DNA damage and fork restarting. To this goal, PANC-1 cells were pulse labeled with IdU for 20 minutes, then treated with 50 nM gemcitabine for 1, 2, 3 and 4 hours, respectively. Cells at each time point were labeled with CIdU for 20 minutes, and collected for neutral comet assay (to examine double-strand DNA damage) and DNA fiber assay (to examine fork restarting) in parallel (Supplementary Fig. 2H). The levels of DNA damage and efficiency of fork restarting were quantified and normalized by the control value (control sample with vehicle but without Gem treatment). In the process of gemcitabine treatment, fork restarting efficiency decreased gradually, while the levels of DNA damage increased dramatically, reminding that fork restarting deficiency could contribute to DNA damage accumulation (Supplementary Fig. 2I). Indeed, when using cells at 4 hours post gemcitabine treatment for immunostaining, we observed dramatic co-localization of CIdU and γH2A.X (Supplementary Fig. 2 J), highlighting DNA damage occurred at fork restarting sites.

We next sought to determine the functions of RETSAT in replication fork dynamics. *RETSAT* deletion was achieved using CRISPR mediated gene knockout technology in PANC-1 and BxPC-3 cells (Fig. 3C). DNA fiber assay is a valuable method to evaluate many aspects of DNA replication at single fork resolution, e.g. fork velocity, nascent DNA stability and stalled fork restart [55]. The treatment of 2.5 mM HU for 4 hours dramatically impaired fork velocity and nascent DNA stability, indicating that the dosage of HU could induce replication stress successfully. We found that *RETSAT* knockout did not change fork velocity (*P* = 0.1377 Supplementary Fig. 2 K) or nascent DNA stability (*P* = 0.2965 Supplementary Fig. 2 L). Then we focused on fork restarting. Under normoxia (21% O_2_), treatment of 2.5 mM HU for 4 hours had no influence on fork restarting in parental cells (*P* = 0.6692 between 1st and 3rd bars), while the same treatment significantly decreased fork restarting under severe hypoxia (0.3% O_2_) (*P* = 0.0019 between 5th and 7th bars, Fig. 3D-E), indicating a synergically detrimental efforts of HU and hypoxia on fork restarting. *RETSAT*-KO cells showed much worse fork restarting under HU treatment (*P* < 0.0001 between 2nd and 4th bars), or severe hypoxia condition (*P* < 0.0001 between 5th and 6th bars). Notably, under co-induced stresses from HU and severe hypoxia, parental cells could maintain fork restarting (*P* = 0.3264 between 3rd and 7th bar), while *RETSAT*-KO cells got further decreased efficiency of fork restart (*P* = 0.0124 between 4th and 8th bar) (Fig. 3E). Together, we concluded RETSAT promotes fork restarting under replication stress.

BLM and SMARCAL1 are two key factors of fork restarting machinery [56, 57]. To investigate the importance of fork restarting system in gemcitabine resistance of PDAC cells, we knocked down these two factors in PANC-1 gemcitabine resistant (PANC-1/Gem-R) line, respectively (Supplementary Fig. 3A-B). Each gene was targeted using two different short hairpin RNAs. Under 50 nM gemcitabine induced replication stress, PANC-1/Gem-R cells were more efficiently to restart stalled forks (*P* < 0.0001, grey region in Supplementary Fig. 3C), and resistant to gemcitabine induced apoptosis (*P* < 0.0001, grey region in Supplementary Fig. 3E) than parental counterpart, emphasizing the correlation of fork restarting abilities and gemcitabine resistance. Consistent to published results [56, 57], knocking down either *BLM* or *SMARCAL1* decreased fork restarting efficiency significantly (yellow region in Supplementary Fig. 3C). Correspondingly, when performing flow cytometry based apoptotic analysis, we found dramatically increased apoptosis in *BLM* or *SMARCAL1* knocking down cells compared to shRNA vector control (*P* < 0.0001, yellow region in Supplementary Fig. 3D-E), indicating that PANC-1/Gem-R cells lost gemcitabine resistance when fork restarting machinery were disturbed. We concluded from these results that fork restarting system is crucial for PDAC cells resistant to gemcitabine.

Persistent stalling forks are prone to transform into DNA breaks consequently, which causes DNA damage accumulation and genomic instability [58]. Consistently, both immunostaining (Fig. 3F-G) and immunoblotting (Fig. 3H-I) using DNA damage marker γH2A.X revealed drastically higher level of DNA breaks in *RETSAT*-KO compared to parental. Notably, almost all γH2A.X foci co-localized with BrdU positive site in *RETSAT*-KO cells, further supporting the DNA breaks were derived from stalled forks predominantly (zoomed panel in Fig. 3F). Consistently, when performing neutral comet assay to evaluate DNA damage, we found that accumulated DNA double strand breaks in *RETSAT*-KO cells under HU and severe hypoxia combined stresses (*P* < 0.0001 between 7th and 8th groups) (Fig. 3J).

Over threatened by replication stress and DNA damage predominantly initiates ATR-CHK1 signaling induces apoptosis [59]. We next sought to find out the determinants behind apoptotic sensitivity of RETSAT knockout cells in response to such stresses. Immunoblotting results revealed that the levels of both active ATR (phosphorylation at serine 428) and active CHK1 (phosphorylation at serine 345) were higher in *RETSAT*-KO cells compared to parental (Fig. 3K), indicating over-activated ATR-CHK1 signaling in *RETSAT*-KO cells. When treated cells with CHK1 antagonist PF-477736 [60](1 μM for 72 hours), we found PF-477736 could dramatically relieve apoptosis in *RETSAT*-KO cells (*P* = 0.0006 Gem and PF-477736 combined group versus Gem single treated group under 0.3% O_2_, Fig. 3L). Consistently, the colony formation ability of *RETSAT*-KO cells was partially rescued as well (Fig. 3M-N). These results indicated that CHK1 signaling promotes apoptosis in *RETSAT* knockout PDAC cells under replication stress.

### RETSAT recruits DDX39B onto replication forks to resolve R-loop

**Fig. 4:**
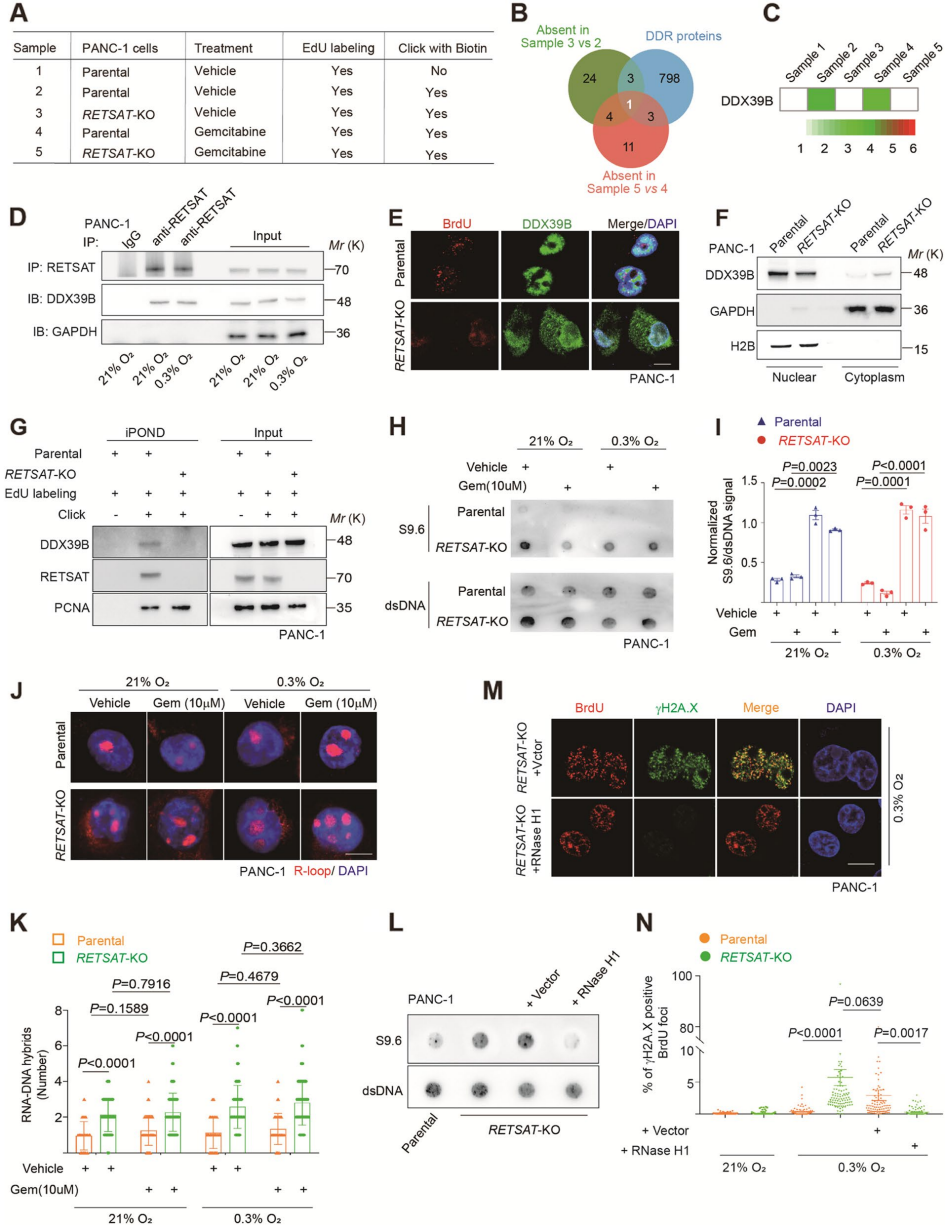
RETSAT recruits DDX39B onto replication forks to resolve R-loop. **A** Treatment and sample collections used for iPOND combined with LC-MS/MS detection. **B** Overlapping analysis between absent proteins in *RETSAT*-KO groups and DNA damage response protein dataset. **C** Heatmap of DDX39B based on its abundance in LC-MS/MS results. **D** Co-immunoprecipitation using anti-RETSAT antibody combined with immunoblotting to confirm the interaction of RETSAT and DDX39B in PANC-1 cells under indicated treatments. GAPDH was included as a negative control. **E** Co-immunostaining of DDX39B (green) and pulse labeled BrdU (red) in parental and *RETSAT*-KO PANC-1 cells. **F** Immunoblotting of DDX39B in nuclear and cytoplasmic extractions of PANC-1 cells with or without RETSAT. GAPDH and H2B were used as a cytoplasmic and nuclear loading control respectively. **G** iPOND combined with immunoblotting to examine DDX39B loading dosage on replication forks in parental and *RETSAT*-KO PANC-1 cells. **H, I** Representative images (**H**) and quantification (**I**) of R-loop dot blotting using S9.6 antibody in parental and *RETSAT*-KO PANC-1 cells under indicated treatments. Anti-dsDNA antibody was used as a loading control. **J, K** Representative images (**J**) and quantification (**K**) of R-loop accumulation in parental and *RETSAT*-KO PANC-1 cells under indicated treatments. **L** Dot Blotting of R-loops using S9.6 antibody in parental and *RETSAT*-KO PANC-1 cells with or without RNase H1 ectopic expression. Anti-dsDNA antibody was used as a loading control. **M, N** Representative images (**M**) and quantification (*N*) of γH2A.X positive BrdU foci in *RETSAT*-KO PANC-1 cells with or without ectopically expression of RNase H1 under indicated cultural conditions. Scale bar = 10 μm. *n* = 3 independent experiments unless otherwise stated. All data are presented as mean ± SEM. *P* values were calculated using a two-tailed student’s *t* test

Based on the functional analysis of RETSAT in fork restarting, we next sought to examine the proteomic changes of replisome with or without RETSAT to find out its molecular mechanisms. To this goal, we performed iPOND combined with LC-MS/MS screening in parental and *RETSAT*-KO PANC-1 cells (Fig. 4A and Supplementary Fig. 4A). The cells were treated with vehicle (DMSO) (sample 2 and 3) or gemcitabine (sample 4 and 5) to induce replication stress. Meanwhile, the parental PANC-1 with EdU labeling but without Biotin click (sample 1) was set up as a non-specific binding control. The proteins identified in sample 1 were defined as non-specific and excluded from the rest samples (Supplementary Table 1). Under normal cultural conditions, we identified 32 proteins missing from *RETSAT*-KO forks and 9 proteins newly emerged in *RETSAT*-KO sample compared to parental (Supplementary Fig. 4B-C and Supplementary Table 2). Under gemcitabine induced replication stress conditions, 19 proteins were absent from in *RETSAT*-KO sample, and 14 proteins were newly emerged (Supplementary Fig. 4D-E and Supplementary Table 3).

We overlapped subgroups of absent proteins in *RETSAT*-KO samples under either normal cultured (32 proteins in Supplementary Fig. 4B) or gemcitabine induced replication stress conditions (19 proteins in Supplementary Fig. 4D). Five proteins including DDX39B, HNRNPA3, RDX, PGK1 and RPL30 were identified as shared missing members in *RETSAT*-KO samples (Supplementary Fig. 4B and D, highlighted in red). Since we have confirmed the functions of RETSAT in DNA replication and genomic stability, we further overlapped these five proteins with the dataset of DNA damage response genes (Supplementary Table 4) (downloaded from http://amigo.geneontology.org). Finally, only one protein named DDX39B was screened out (Fig. 4B-C).

DDX39B (also named as UAP56 or BAT1) is a DEAD-box family helicase and plays pivotal roles in mRNA binding, splicing, and export [61]. In the process of DNA replication, DDX39B is responsible for unwinding R-loops to avoid collisions between DNA replication machinery and unresolved R-loops, finally save genomic stability [22, 62]. We first performed co-immunostaining assay to examine the location of R-loop and DNA damage sites. As shown in Supplementary Fig. 5A, significant co-localization of R-loop and γH2A.X foci in PANC-1 cells under gemcitabine treatment. When ectopically expressed RNase H1 in PANC-1 cells (Supplementary Fig. 5B), the overall DNA damage levels in gemcitabine treated group was downregulated dramatically (Supplementary Fig. 5C-D), highlighting R-loop is involved in gemcitabine induced DNA damage.

To investigate the functions of DDX39B in gemcitabine resistance, we knocked down *DDX39B* in PANC-1/Gem-R cells. Western blotting was performed to confirm knocking down efficiency (Supplementary Fig. 5E). Compared with shRNA vector control, the capacities of in vitro proliferation (Supplementary Fig. 5F-G) and colony formation (Supplementary Fig. 5H-I) were dramatically decreased in PANC-1/Gem-R cells. Notably, PANC-1/Gem-R cells without efficient DDX39B expression were sensitive to gemcitabine induced apoptosis (Supplementary Fig. 5 J). These results highlighted the importance of DDX39B in gemcitabine resistance of pancreatic cancer cells.

We isolated nuclear protein lysis of PANC-1 cells and confirmed the interaction of RETSAT and DDX39B under gemcitabine treatment or severe hypoxia using co-immunoprecipitation assay (Fig. 4D and Supplementary Fig. 5 K). Immunoblotting results revealed that *RETSAT* knockout did not change the total abundance of DDX39B, and *vise versa* (Supplementary Fig. 5 L-M). When examining subcellular localizations, we found that *DDX39B* knocking down did not change the location of RETSAT onto replication foci (Supplementary Fig. 5 N). Notably, DDX39B locates in nuclear in PANC-1 parental cells (upper panel in Fig. 4E), while we observed significant amounts of DDX39B released into cytoplasm in *RETSAT*-KO cells (lower panel in Fig. 4E). We further validated this phenotype using cytoplasm-nuclei separation kit and immunoblotting assay. The results showed that nuclear DDX39B was decreased in *RETSAT*-KO cells, while cytoplasmic DDX39B was dramatically increased correspondingly (Fig. 4F), further supporting our observation in immunofluorescence assay (Fig. 4E). DDX39B functions to resolve R-loops on the whole chromatin level [22]. Especially, we confirmed the loading of DDX39B on forks was dramatically decreased without RETSAT by using iPOND assay (Fig. 4G).

To validate fork restarting defects in *RETSAT*-KO cells was caused by R-loop accumulation, we performed R-loop dot blotting (Fig. 4H-I) and immunofluorescence (Fig. 4J-K) using S9.6 antibody, and confirmed overwhelming R-loops in *RETSAT*-KO PANC-1 cells. When ectopically expressed RNase H1 in *RETSAT*-KO cells (Supplementary Fig. 5O), the overwhelmed R-loops were efficiently resolved in *RETSAT*-KO cells (Fig. 4L), although abundant DDX39B still existed in cytoplasm (Supplementary Fig. 5P). As such, we observed dramatically decrease of γH2AX positive replication foci in *RETSAT*-KO cells even under severe hypoxia (Fig. 4M-N). Together, we concluded that RETSAT is responsible for recruitment of DDX39B onto forks, through which resolves R-loop obstacle and saves fork stability.

### Evaluation of synergetic effects of DDX39B inhibitor CCT018159 and gemcitabine in human PDAC organoids system

**Fig. 5:**
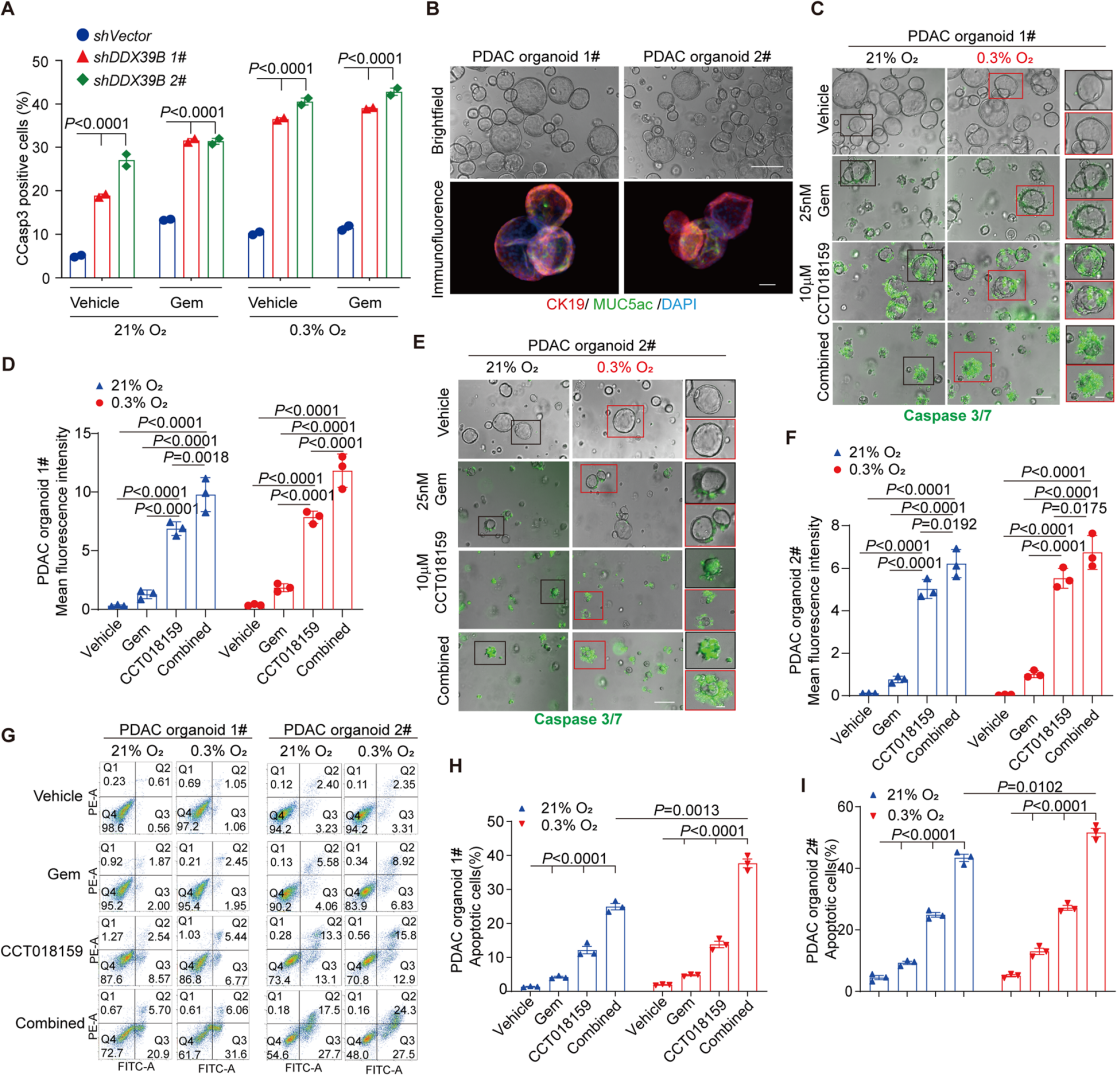
Synergetic evaluation of DDX39B inhibitor CCT018159 and gemcitabine in human PDAC organoids system. **A** Apoptosis of PANC-1 cells with or without DDX39B knocking down under indicated treatments. **B** Brightfield morphology and immunostaining of CK19 and MUC5AC in PDAC organoids. **C, D** Representative images (**C**) and quantification (**D**) of apoptosis using green-fluorescent caspase 3/7 probe labeling in PDAC organoids 1# under indicated treatments. 25 nM Gemcitabine and 10 μM CCT018159 were used single or combined. **E, F** Representative images (**E**) and quantification (**F**) of apoptosis using green-fluorescent caspase 3/7 probe labeling in PDAC organoids 2# under indicated treatments. 25 nM Gemcitabine and 10 μM CCT018159 were used single or combined. The green fluorescence was observed with fluorescence microscope, the density of fluorescence was quantified with Image J software. **G-I** Representative images (**G**) and quantification (**H, I**) of apoptotic comparison using FITC Annexin V marked flow cytometry analysis of PDAC organoids 1# and 2# under indicated treatments. **J** Graphical model of this study. Scale bar = 200 μm in top panel and 100 μm in bottom panel in (**B**), 200 μm in left two panel and 100 μm in right panel in (**C**) and (**E**). *n* = 3 independent experiments unless otherwise stated. All data are presented as mean ± SEM. *P* values were calculated using a two-tailed student’s *t* test

The association of RETSAT and DDX39B is crucial for fork restarting and genomic stability. Knocking out *RETSAT* in PANC-1 sensitized cells to gemcitabine induced apoptosis (Fig. 2J). Notably, knocking down *DDX39B* exhibited similar apoptotic phenotype under either 20% or 0.3% oxygen tensions (Fig. 5A, Supplementary Fig. 5 M), highlighting the two proteins to be druggable targets for PDAC chemotherapy.

DDX39B unwinds R-loop relying on its ATPase activity, because it has been confirmed that DDX39B -K95A and -E197A mutants that defective for the ATPase activity could not unwind R-loop [22]. Notably, CCT018159, originally identified as a heat shock protein 90 (HSP90) inhibitor [63], was found to be able to inhibit the ATPase activity of DDX39B in antiviral study [64]. We wondered the possibility of CCT018159 in PDAC chemotherapy. To this goal, we derived two PDAC organoid lines from surgery tumor tissues following standard protocol (Fig. 5B) [46–48]. The organoids were treated with vehicle, 25 nM gemcitabine, 10 μM CCT018159 or combined together and cultured under 20% or 0.3% oxygen tensions for 72 hours. Organoids were labeled with green-fluorescent caspase 3/7 probe (Fig. 5C and E), or stained with FITC Annexin V for flow cytometry analysis to evaluate apoptosis (Fig. 5G). In both organoid lines, CCT018159 performed better than gemcitabine under both oxygen tensions (*P* < 0.0001 Gem versus CCT018159 Fig. 5D, F, H, I). Combined treatment showed significantly synergistic effects, with statistical significance and much stronger green fluorescence in combined groups (Fig. 5D, F, H, I).

## Discussion

Here we report that *RETSAT* gene plays key roles in TME hypoxia adaptation and gemcitabine chemotherapy in the context of pancreatic ductal carcinoma (PDAC). Our study demonstrates that RETSAT is a fork associated protein in the nuclear. HIF-1α signaling promotes the expression of RETSAT upstream. RETSAT interacts with DDX39B, and recruits DDX39B onto replication forks to resolve R-loops and avoids collisions between DNA replication and transcription machineries, through which saves fork restarting and avoids fork damage initiated CHK1 activation and apoptosis. However, there are a few limitations in our study. Although we focus on the nuclear functions of RETSAT, RETSAT has been well defined as an oxidoreductase in the cytoplasm that catalyzes retinol into 13,14-dihydroretinol, we cannot formally exclude the possibility that other mechanisms may also directly or indirectly contribute to the phenotypes of this study. Additionally, we did not confirm the interactions of RETSAT with other proteins we identified through iPOND-MS. We studied the synergic effects of CCT018159 and gemcitabine in PDAC organoids system. However, CCT018159 is developed to be an antagonist of HSP90. Recently, it was revealed that CCT018159 has inhibition effects to DDX39B. So CCT018159 is not a selective antagonist targeting DDX39B. Meanwhile, HSP90 has been reported to play multiple roles in pancreatic cancers, e.g. chromosome stability [65], JAK/STAT and MAPK signaling [66], we did not exclude the participation of HSP90 in our study. Regardless, this dataset demonstrates that enhancing fork damage and CHK1 signaling through targeting R-loop helicase can be explored for sensitizing pancreatic cells to gemcitabine. We anticipate our findings to have far-reaching implications for developing future combinatory therapeutics of pancreatic cancer. So far, there is no selective antagonists available targeting RETSAT or DDX39B. To achieve this goal, drug development targeting RETSAT and DDX39B specifically will be key works needed to be addressed.

Our immunofluorescence results revealed tremendous co-localization of RETSAT and BrdU pulse labeled replication foci. However, in the peptide list identified from iPOND combined with mass spectrometry analysis, we got no RETSAT peptides. When performing iPOND combined with immunoblotting to detect RETSAT, we found it was uneasy to detected RETSAT following standard iPOND procedure. We had to synchronize cells into S phase in order to purify replication forks as many as possible, then we were able to detect bands of RETSAT in immunoblotting. Based on our experiences in the previous study, classical fork binding proteins such as PCNA or RPAs could be easily detected in iPOND assay [40]. Compared with them, the abundance of RETSAT on replication forks might be low. This phenomenon might be useful to understand the biological characters of RETSAT protein more detailedly.

In our previous study, we have identified *RETSAT* as a convergent gene in high-altitude mammal species, emphasizing the contribution of *RETSAT* in mammalian hypoxia adaptation [32]. Here we report its functions in pancreatic cancer cells. This indicates the possible conservation of hypoxia adaptation between high-altitude mammalians and solid tumor cells. Our study demonstrated that translating mammalian genetic resources in high-altitude adaption into oncological hypoxia research might be an alternative avenue towards precision tumor therapy.

## Conclusions

**Fig. 6:**
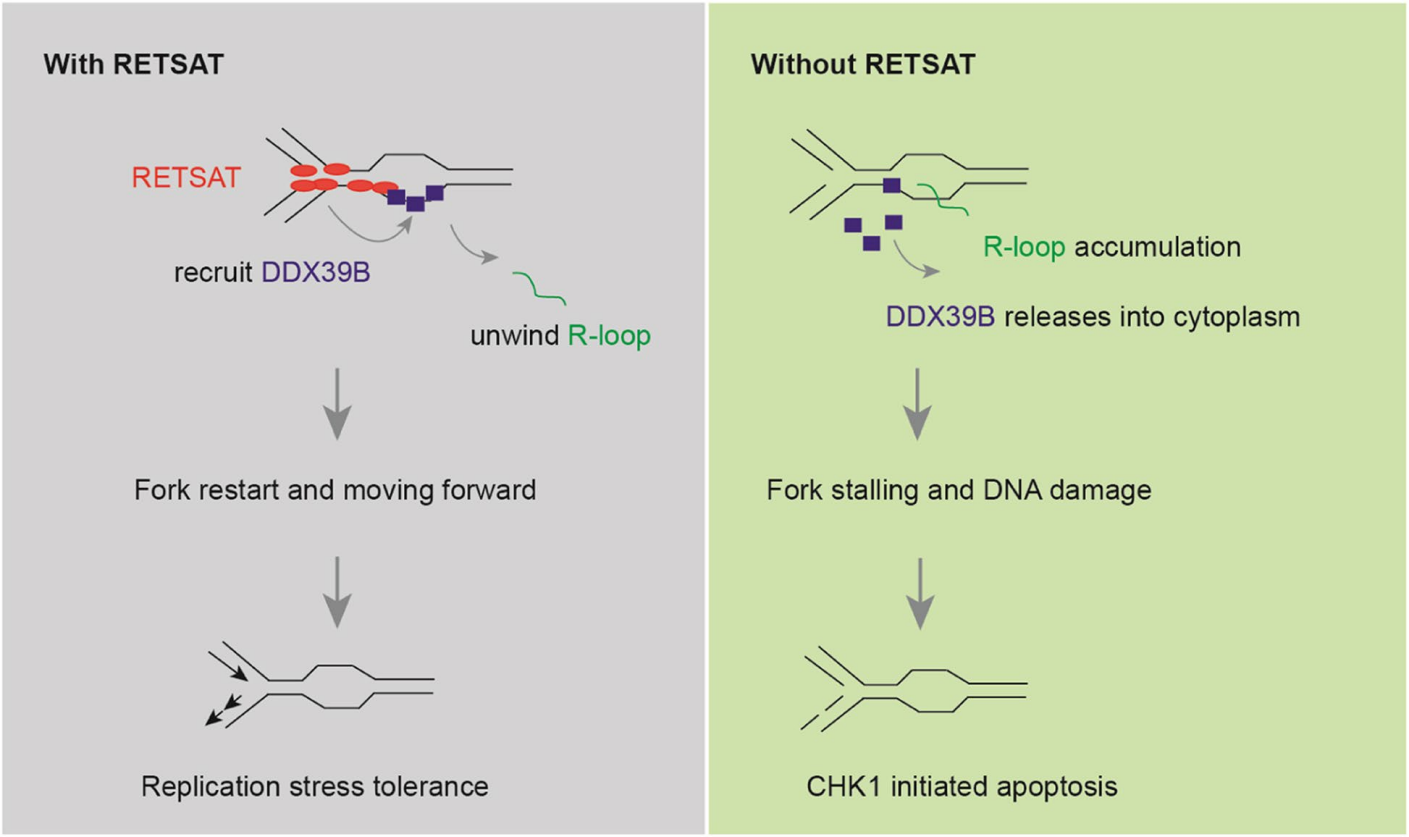
Graphic abstract of the study

In this study, we identified RETSAT to be a novel replication fork protein. Hypoxia upregulates RETSAT expression. RETSAT interacts with DDX39B in order to resolve R-loops and avoid collisions occurred between replication forks and transcriptional machinery, through which finally promotes fork restarting and endows PDAC cells resistant to gemcitabine chemotherapy. Our study highlighted the importance of RETSAT mediated fork restarting mechanisms in hypoxia adaptation and gemcitabine resistance of PDAC, and provided CCT018159 to be a useful chemical in PDAC chemotherapy. In summary, these findings shed light on novel molecular mechanisms and provide new insight into developing effective therapeutic strategies for pancreatic ductal adenocarcinoma (Fig. 6).

## Supplementary Information

**Additional file 1: Supplementary Fig. 1.** *RETSAT* deletion sensitizes PDAC cells to gemcitabine. (*A*) The expression of *RETSAT* in *KRAS* mutant (*n* = 86) and *KRAS* wild type (*n* = 10) PDAC tumor tissues from TCGA database. (*B, C*) Immunostaining (*B*) and quantification (*C*) of cell proliferation marker Ki67 in parental and *RETSAT*-KO PANC-1 cells with or without gemcitabine treatment. (*D, E*) Immunostaining of cleaved caspase 3 (*D*) and flow cytometry based Annexin V apoptosis quantification (*E*) in parental and *RETSAT*-KO PANC-1 cells with or without gemcitabine treatment under 21% O_2_ or 0.3% O_2_. (*F*, *G*) Images (*F*) and quantification (*G*) of flow cytometry based Annexin V apoptosis of 3D culture PANC-1 spheroids under indicated treatments. (*H*, *I*) Images (*H*) and quantification (*I*) of in vivo bioluminescence of all mice at indicated time. (*J*-*M*) Bioluminescence quantifications of each group including parental with Vehicle (*J*), *RETSAT*-KO with Vehicle (*K*), parental with Gem (*L*), *RETSAT*-KO with Gem (*M*) were shown. Scale bar = 100 μm. *n* = 3 independent experiments unless otherwise stated. All data are presented as mean ± SEM. *P* values were calculated using a two-tailed student’s *t* test. **Supplementary Fig. 2.** RETSAT localizes onto DNA replication forks and has no effects on fork velocity or nascent DNA stability. (*A*) Immunofluorescence of RETSAT in PANC-1 cells with or without 0.2% Triton X-100 pre-wash ahead of paraformaldehyde fixation. (*B*) Co-immunostaining of RETSAT and telomeric PNA probe in BxPC-3 cells. (*C*, *D*) Co-immunostaining of RETSAT (green) and BrdU pulse labeled replication foci (red) in BxPC-3 and PANC-1 cells cultured under vehicle or HU induced stress conditions. (*E*) Co-immunostaining of RETSAT (green) and BrdU pulse labeled replication foci (red) in BxPC-3 cells under 21% or 0.3% O_2_ conditions. (*F*, *G*) Immunoblotting of RETSAT in PANC-1 cells treated with 4 mM HU (*F*) or 10 μM gemcitabine (*G*) at indicated time points. β actin was used as a loading control. (*H, I*) Experimental setup (*H*) and quantifications (*I*) of DNA fiber and neutral comet assay in PANC-1 cells treated with 50 nM gemcitabine at indicated time points. (*J*) Co-immunostaining of γH2A.X (green) and CIdU labeled fork restarting sites (red) in PANC-1 cells treated with 50 nM gemcitabine. (*K*) Quantification of fork velocity in parental and *RETSAT*-KO PANC-1 cells with or without HU treatment. At least 200 single forks were calculated in each sample. (*L*) Evaluation of nascent DNA stability in parental and *RETSAT*-KO PANC-1 cells with or without HU treatment, calculated by ratio of CIdU length divided by own IdU length. At least 200 single forks were calculated in each sample. Scale bar = 10 μm. *n* = 3 independent experiments unless otherwise stated. All data are presented as mean ± SEM. *P* values were calculated using a two-tailed student’s *t* test. **Supplementary Fig. 3.** Fork restarting system is crucial for PDAC cells resistant to gemcitabine. (*A, B*) Immunoblotting of BLM (*A*) and SMARCAL1 (*B*) in PANC-1/Gem-R cells transfected with shVector or indicated shRNA lentivirus. β actin was used as a loading control. (*C*) Quantification of fork restarting in PANC-1 and PANC-1/Gem-R cells with or without *BLM* and *SMARCAL1* knocking down under vehicle or 50 nM gemcitabine treatment. (*D, E*) Images (*D*) and quantification (*E*) of flow cytometry based Annexin V apoptosis of PANC-1 and PANC-1/Gem-R with or without *BLM* and *SMARCAL1* knocking down under vehicle or 10 μM gemcitabine treatment. *n* = 3 independent experiments unless otherwise stated. All data are presented as mean ± SEM. *P* values were calculated using a two-tailed student’s *t* test. **Supplementary Fig. 4.** Changes of replisome components in response to *RETSAT* knocking out using iPOND combined with LC-MS/MS identification. (*A*) Corresponding to Fig. 4A, schematic of iPOND assay combined with LC-MS/MS analysis. (*B*) Heatmap of absent proteins in *RETSAT*-KO PANC-1 cells compared with parental under vehicle treatment. (*C*) Heatmap of newly emerged proteins in *RETSAT*-KO PANC-1 cells compared with parental under vehicle treatment. (*D*) Heatmap of absent proteins in *RETSAT*-KO PANC-1 cells compared with parental under gemcitabine treatment. (*E*) Heatmap of newly emerged proteins in *RETSAT*-KO PANC-1 cells compared with parental under gemcitabine treatment. **Supplementary Fig. 5.** RETSAT interacts with DDX39B and avoids R-loop accumulation. (*A*) Co-immunostaining of γH2A.X (green) and R-loop (red) in PANC-1 cells with or without 10 μM gemcitabine treatment. S9.6 antibody was used to label R-loop. (*B*) Immunoblotting of RNase H1 in PANC-1 cells with or without RNase H1 ectopic expression. β actin was used as a loading control. (*C*, *D*) Immunostaining (*C*) and quantification (*D*) of γH2A.X (green) positive PANC-1 cells with or without RNase H1 ectopic expression under indicated treatment. (*E*) Immunoblotting of DDX39B in PANC-1/Gem-R cells with or without *DDX39B* knocking down. (*F*, *G*) Immunostaining (*F*) and quantification (*G*) of Ki67 in PANC-1/Gem-R cells with or without *DDX39B* knockdown under vehicle or 10 μM gemcitabine treatment. (*H*, *I*) Clone formation (*H*) and quantification (*I*) of PANC-1/Gem-R cells with or without *DDX39B* knocking down under vehicle or 10 μM gemcitabine treatment. (*J*) Flow cytometry based Annexin V apoptotic analysis of PANC-1/Gem-R cells with or without *DDX39B* knockdown under vehicle or 10uM gemcitabine treatment. (*K*) Co-immunoprecipitation using anti-RETSAT antibody combined with immunoblotting to confirm the interaction of RETSAT and DDX39B in PANC-1 cells under vehicle or gemcitabine treatments. GAPDH was used as a negative control. (*L*, *M*) Immunoblotting of RETSAT and DDX39B in parental, *RETSAT*-KO and *DDX39B* knocking down PANC-1 cells. β actin was used as a loading control. (*N*) Co-immunostaining of RETSAT (green) and BrdU pulse labeled replication foci (red) in PANC-1 cells with or without DDX39B under indicated conditions. (*O*) Immunoblotting of RNase H1 in *RETSAT*-KO PANC-1 cells with or without RNase H1 ectopic expression. β actin was used as a loading control. (*P*) Immunostaining of DDX39B in parental and *RETSAT*-KO PANC-1 cells with or without RNase H1 ectopic expression. Scale bar = 10 μm in (*A*) and (*H*), 50 μm in (*C*) and (*F*). *n* = 3 independent experiments unless otherwise stated. All data are presented as mean ± SEM. *P* values were calculated using a two-tailed student’s *t* test.

**Additional file 2.**

**Additional file 3.**

**Additional file 4.**

**Additional file 5.**

## Data Availability

Mass spectrometry data is available at Proteome Xchange, Project accession: PXD032951. The data sets used and/or analyzed during the current study are available from the corresponding author on reasonable request.

## Abbreviations

PDAC: Pancreatic ductal adenocarcinoma
PANC-1/Gem-R: Gemcitabine resistant PANC-1 subline cell
CDX: Cell derived xenograft
KO: Knockout
T-FISH: Telomeric fluorescence in situ hybridization
iPOND: Isolate proteins on nascent DNA
HU: Hydroxyurea
Gem: Gemcitabine
TME: Tumor microenvironment
RNR: Ribonucleotide reductase
BrdU: Bromodeoxyuridine
EdU: 5-ethynyl-2′-deoxyuridine
IdU: 5-Iodo-2-deoxyuridine
CIdU: 5-chloro-2′-deoxyuridine
HCl: Hydrochloric acid
EDTA: Ethylene diamine tetraacetic acid
PBS: Phosphate buffer solution
DMSO: Dimethylsulfoxide
BSA: Bovine serum albumin
SDS: Sodium dodecyl sulfate
HPLC: High performance liquid chromatography
FDR: False discovery rates
Co-IP: Co-immunoprecipitation
TCGA: The Cancer Genome Atlas
IHC: Immunohistochemistry
FPKM: Fragments per kilobase million
qRT-PCR: Quantitative real-time PCR
CCasp3: Cleaved caspase 3
LC-MS/MS: Liquid Chromatograph Mass Spectrometer
BLM: Blooms Syndrome
SMARCAL1: SWI/SNF related, matrix associated, actin dependent regulator of chromatin, subfamily a like 1
