# Supplementary material for: RETSAT associates with DDX39B to promote fork restarting and resistance to gemcitabine based chemotherapy in pancreatic ductal adenocarcinoma
Source: J Exp Clin Cancer Res. 2022 Sep 15;41:274. doi: 10.1186/s13046-022-02490-3 (PMC9476698; doi:10.1186/s13046-022-02490-3)

**Supplementary Figure 1** *RETSAT* deletion sensitizes PDAC cells to gemcitabine. (*A*) The expression of *RETSAT* in *KRAS* mutant (n=86) and *KRAS* wild type (n=10) PDAC tumor tissues from TCGA database. (*B, C*) Immunostaining (*B*) and quantification (*C*) of cell proliferation marker Ki67 in parental and *RETSAT*-KO PANC-1 cells with or without gemcitabine treatment. (*D, E*) Immunostaining of cleaved caspase 3 (*D*) and flow cytometry based Annexin V apoptosis quantification (*E*) in parental and *RETSAT*-KO PANC-1 cells with or without gemcitabine treatment under 21% O_2_ or 0.3% O_2_. (*F*, *G*) Images (*F*) and quantification (*G*) of flow cytometry based Annexin V apoptosis of 3D culture PANC-1 spheroids under indicated treatments. (*H*, *I*) Images (*H*) and quantification (*I*) of *in vivo* bioluminescence of all mice at indicated time. (*J*-*M*) Bioluminescence quantifications of each group including parental with Vehicle (*J*), *RETSAT*-KO with Vehicle (*K*), parental with Gem (*L*), *RETSAT*-KO with Gem (*M*) were shown. Scale bar = 100 μm. n = 3 independent experiments unless otherwise stated. All data are presented as mean ± SEM. *P* values were calculated using a two-tailed student's *t* test.

**Supplementary Figure 2** RETSAT localizes onto DNA replication forks and has no effects on fork velocity or nascent DNA stability. (*A*) Immunofluorescence of RETSAT in PANC-1 cells with or without 0.2% Triton X-100 pre-wash ahead of paraformaldehyde fixation. (*B*) Co-immunostaining of RETSAT and telomeric PNA probe in BxPC-3 cells. (*C*, *D*) Co-immunostaining of RETSAT (green) and BrdU pulse labeled replication foci (red) in BxPC-3 and PANC-1 cells cultured under vehicle or HU induced stress conditions. (*E*) Co-immunostaining of RETSAT (green) and BrdU pulse labeled replication foci (red) in BxPC-3 cells under 21% or 0.3% O_2_ conditions. (*F*, *G*) Immunoblotting of RETSAT in PANC-1 cells treated with 4mM HU (*F*) or 10 μM gemcitabine (*G*) at indicated time points. β actin was used as a loading control. (*H, I*) Experimental setup (*H*) and quantifications (*I*) of DNA fiber and neutral comet assay in PANC-1 cells treated with 50nM gemcitabine at indicated time points. (*J*) Co-immunostaining of γH2A.X (green) and CIdU labeled fork restarting sites (red) in PANC-1 cells treated with 50nM gemcitabine. (*K*) Quantification of fork velocity in parental and *RETSAT*-KO PANC-1 cells with or without HU treatment. At least 200 single forks were calculated in each sample. (*L*) Evaluation of nascent DNA stability in parental and *RETSAT*-KO PANC-1 cells with or without HU treatment, calculated by ratio of CIdU length divided by own IdU length. At least 200 single forks were calculated in each sample. Scale bar = 10 μm. n = 3 independent experiments unless otherwise stated. All data are presented as mean ± SEM. *P* values were calculated using a two-tailed student's *t* test.

**Supplementary Figure 3** Fork restarting system is crucial for PDAC cells resistant to gemcitabine. (*A, B*) Immunoblotting of BLM (*A*) and SMARCAL1 (*B*) in PANC-1/Gem-R cells transfected with shVector or indicated shRNA lentivirus. β actin was used as a loading control. (*C*) Quantification of fork restarting in PANC-1 and PANC-1/Gem-R cells with or without *BLM* and *SMARCAL1* knocking down under vehicle or 50nM gemcitabine treatment. (*D, E*) Images (*D*) and quantification (*E*) of flow cytometry based Annexin V apoptosis of PANC-1 and PANC-1/Gem-R with or without *BLM* and *SMARCAL1* knocking down under vehicle or 10μM gemcitabine treatment. n = 3 independent experiments unless otherwise stated. All data are presented as mean ± SEM. *P* values were calculated using a two-tailed student's *t* test.

**Supplementary Figure 4** Changes of replisome components in response to *RETSAT* knocking out using iPOND combined with LC-MS/MS identification. (*A*) Corresponding to Figure 4A, schematic of iPOND assay combined with LC-MS/MS analysis. (*B*) Heatmap of absent proteins in *RETSAT*-KO PANC-1 cells compared with parental under vehicle treatment. (*C*) Heatmap of newly emerged proteins in *RETSAT*-KO PANC-1 cells compared with parental under vehicle treatment. (*D*) Heatmap of absent proteins in *RETSAT*-KO PANC-1 cells compared with parental under gemcitabine treatment. (*E*) Heatmap of newly emerged proteins in *RETSAT*-KO PANC-1 cells compared with parental under gemcitabine treatment.

**Supplementary Figure 5** RETSAT interacts with DDX39B and avoids R-loop accumulation. (*A*) Co-immunostaining of γH2A.X (green) and R-loop (red) in PANC-1 cells with or without 10μM gemcitabine treatment. S9.6 antibody was used to label R-loop. (*B*) Immunoblotting of RNase H1 in PANC-1 cells with or without RNase H1 ectopic expression. β actin was used as a loading control. (*C*, *D*) Immunostaining (*C*) and quantification (*D*) of γH2A.X (green) positive PANC-1 cells with or without RNase H1 ectopic expression under indicated treatment. (*E*) Immunoblotting of DDX39B in PANC-1/Gem-R cells with or without *DDX39B* knocking down. (*F*, *G*) Immunostaining (*F*) and quantification (*G*) of Ki67 in PANC-1/Gem-R cells with or without *DDX39B* knockdown under vehicle or 10μM gemcitabine treatment. (*H*, *I*) Clone formation (*H*) and quantification (*I*) of PANC-1/Gem-R cells with or without *DDX39B* knocking down under vehicle or 10μM gemcitabine treatment. (*J*) Flow cytometry based Annexin V apoptotic analysis of PANC-1/Gem-R cells with or without *DDX39B* knockdown under vehicle or 10uM gemcitabine treatment. (*K*) Co-immunoprecipitation using anti-RETSAT antibody combined with immunoblotting to confirm the interaction of RETSAT and DDX39B in PANC-1 cells under vehicle or gemcitabine treatments. GAPDH was used as a negative control. (*L*, *M*) Immunoblotting of RETSAT and DDX39B in parental, *RETSAT*-KO and *DDX39B* knocking down PANC-1 cells. β actin was used as a loading control. (*N*) Co-immunostaining of RETSAT (green) and BrdU pulse labeled replication foci (red) in PANC-1 cells with or without DDX39B under indicated conditions. (*O*) Immunoblotting of RNase H1 in *RETSAT*-KO PANC-1 cells with or without RNase H1 ectopic expression. β actin was used as a loading control. (*P*) Immunostaining of DDX39B in parental and *RETSAT*-KO PANC-1 cells with or without RNase H1 ectopic expression. Scale bar = 10 μm in (*A*) and (*H*), 50 μm in (*C*) and (*F*). n = 3 independent experiments unless otherwise stated. All data are presented as mean ± SEM. *P* values were calculated using a two-tailed student's *t* test.


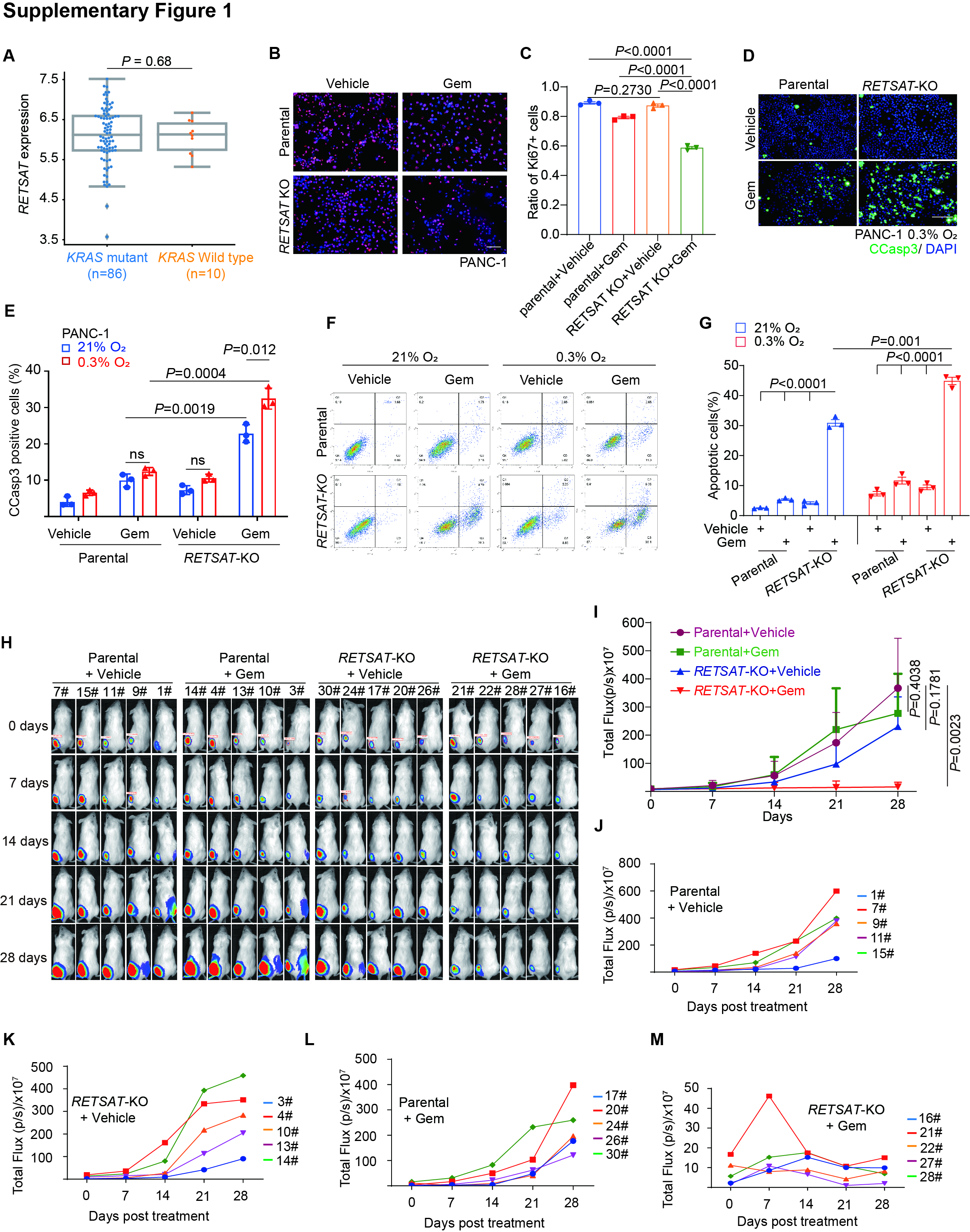


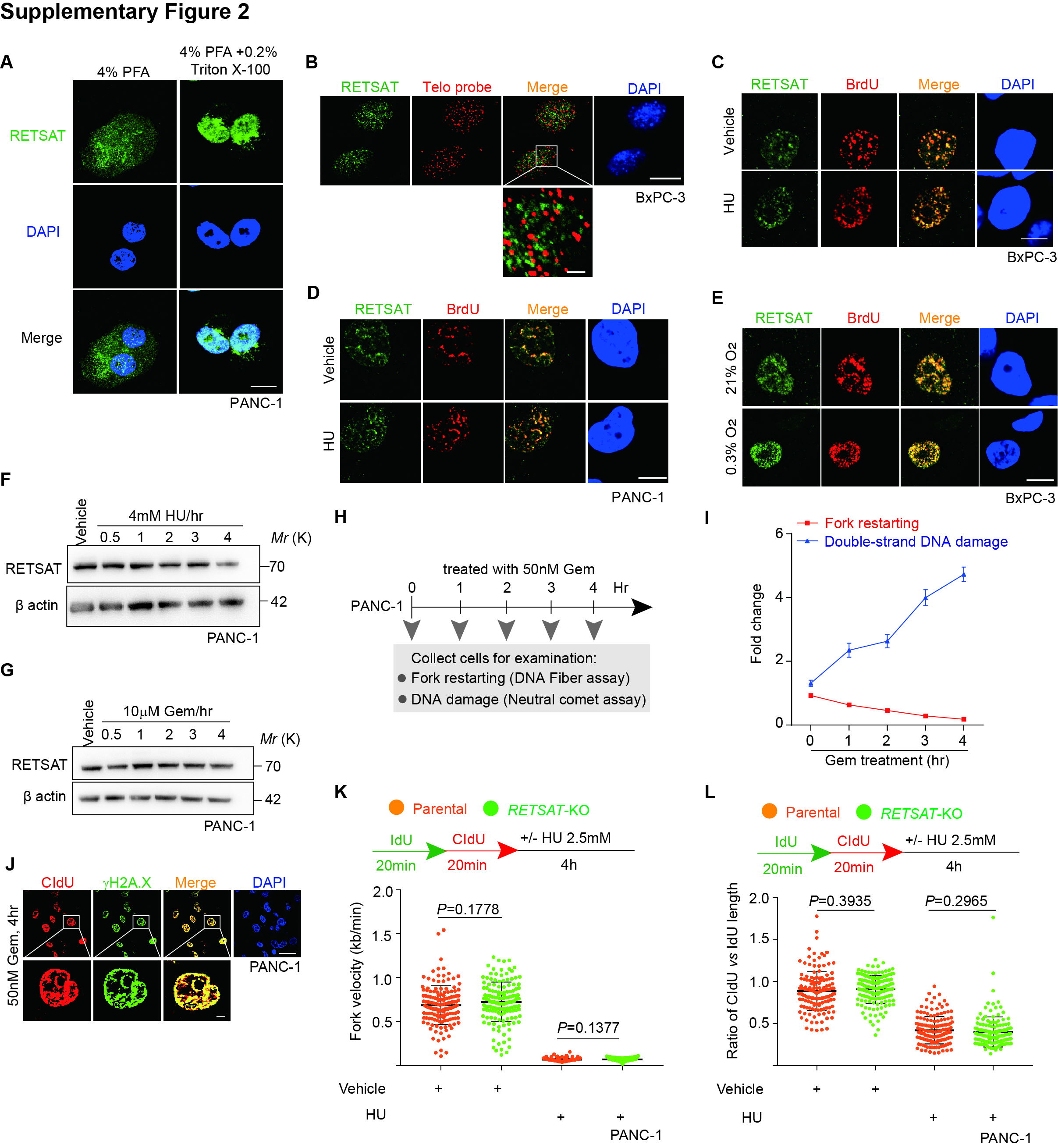


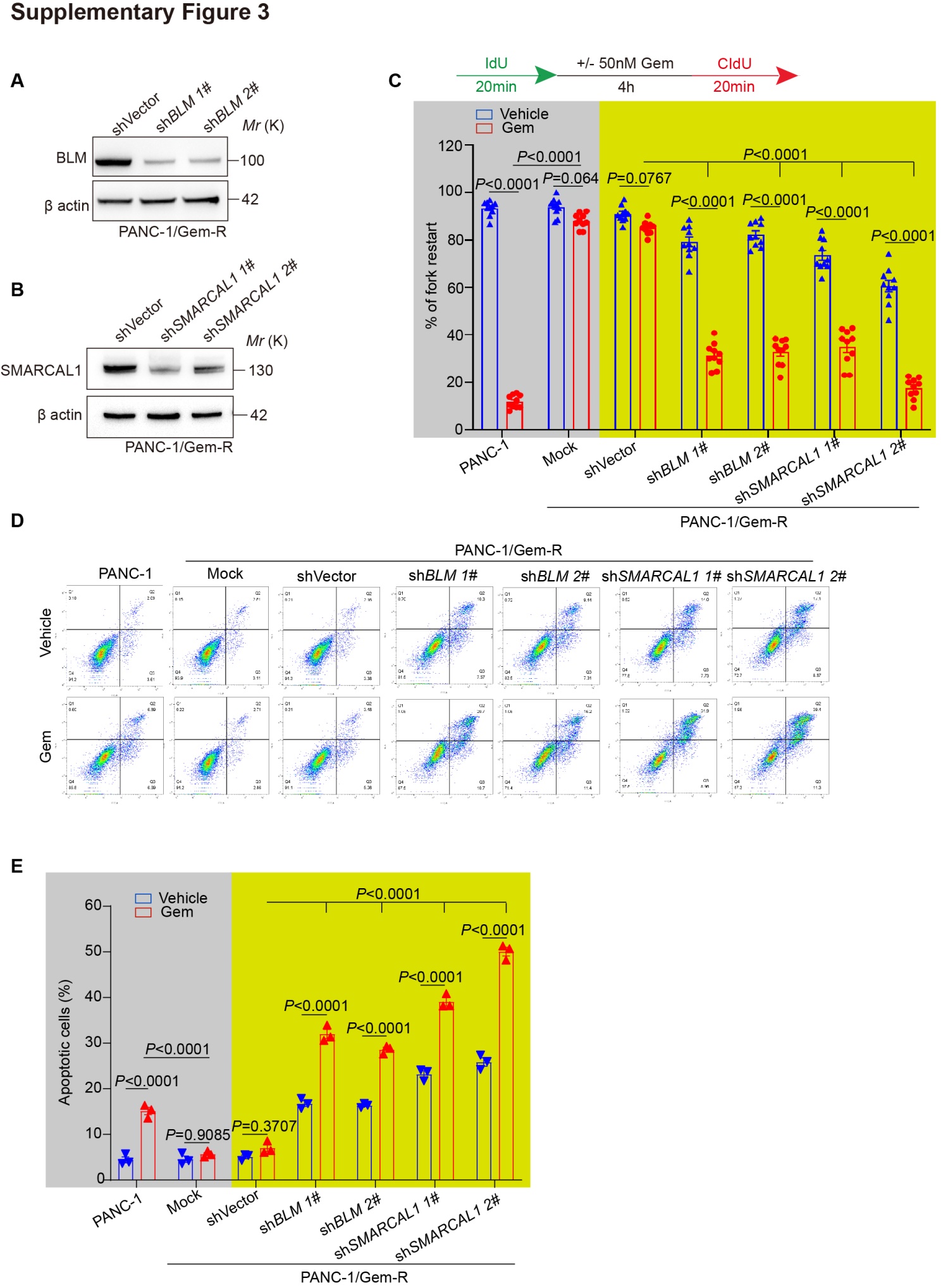


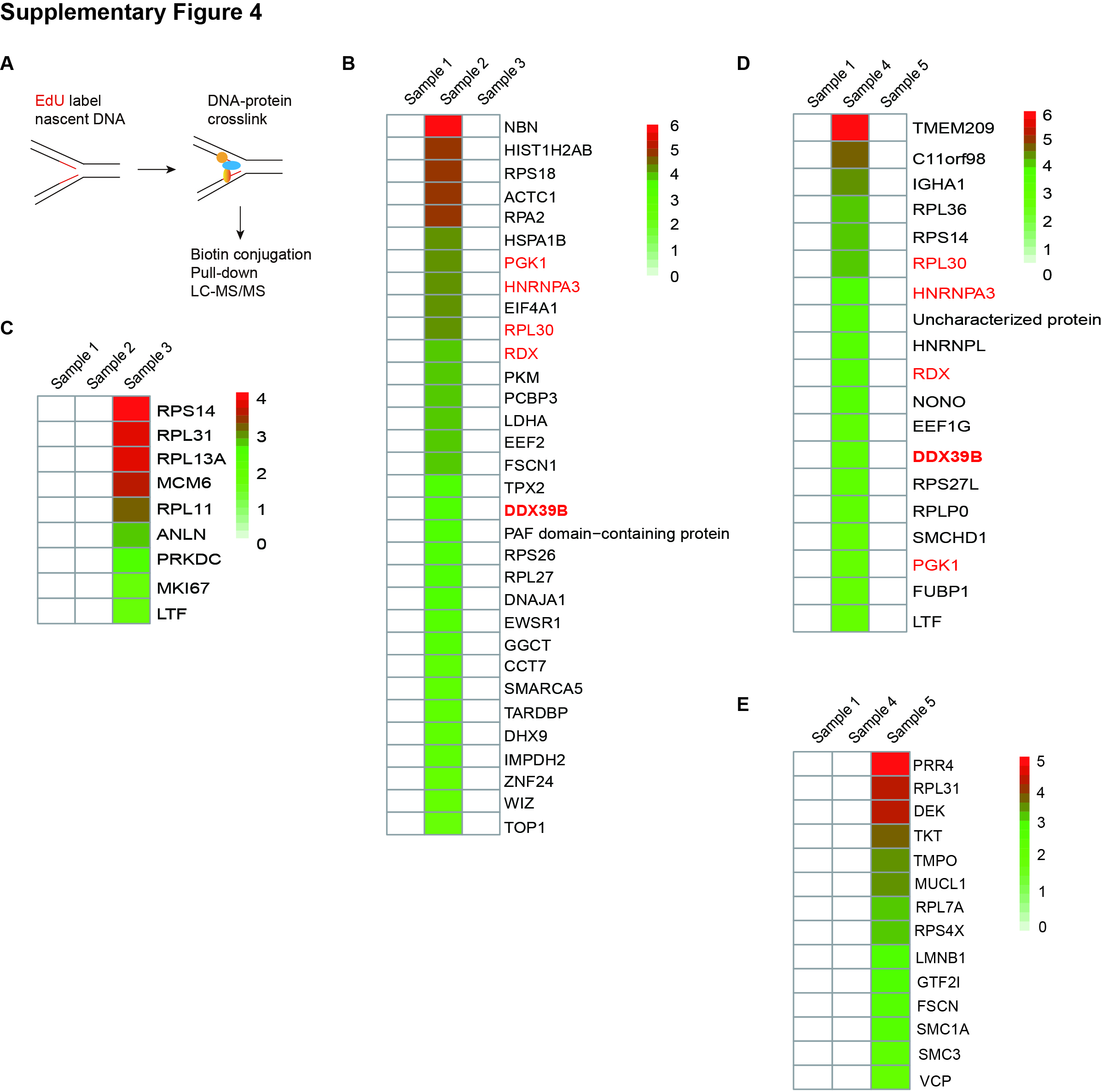


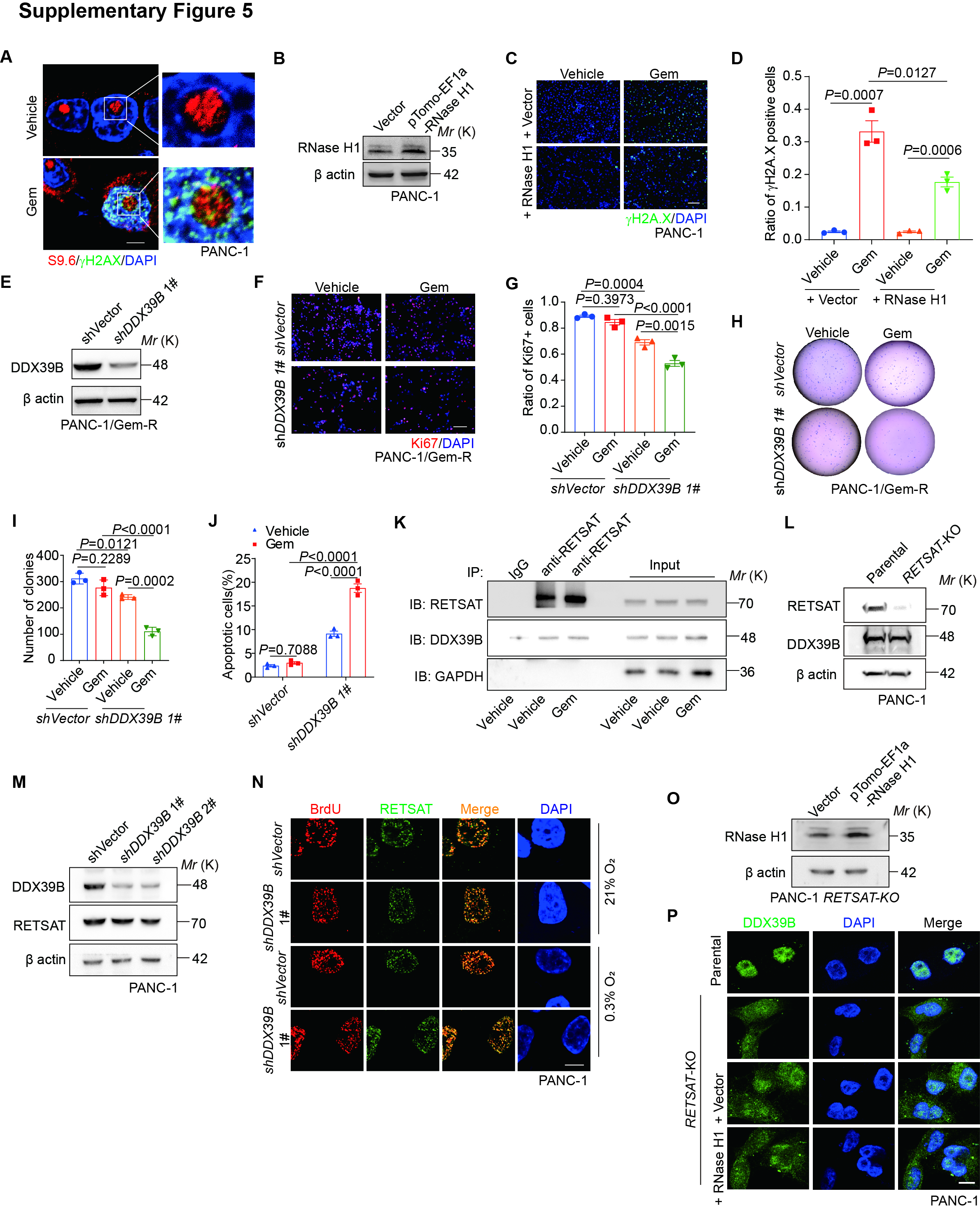

Supplement: Supplementary file 1 — Additional file 1: Supplementary Fig. 1. RETSAT deletion sensitizes PDAC cells to gemcitabine. (A) The expression of RETSAT in KRAS mutant (n = 86) and KRAS wild type (n = 10) PDAC tumor tissues from TCGA database. (B, C) Immunostaining (B) and quantification (C) of cell proliferation marker Ki67 in parental and RETSAT-KO PANC-1 cells with or without gemcitabine treatment. (D, E) Immunostaining of cleaved caspase 3 (D) and flow cytometry based Annexin V apoptosis quantification (E) in parental and RETSAT-KO PANC-1 cells with or without gemcitabine treatment under 21% O2 or 0.3% O2. (F, G) Images (F) and quantification (G) of flow cytometry based Annexin V apoptosis of 3D culture PANC-1 spheroids under indicated treatments. (H, I) Images (H) and quantification (I) of in vivo bioluminescence of all mice at indicated time. (J-M) Bioluminescence quantifications of each group including parental with Vehicle (J), RETSAT-KO with Vehicle (K), parental with Gem (L), RETSAT-KO with Gem (M) were shown. Scale bar = 100 μm. n = 3 independent experiments unless otherwise stated. All data are presented as mean ± SEM. P values were calculated using a two-tailed student’s t test. Supplementary Fig. 2. RETSAT localizes onto DNA replication forks and has no effects on fork velocity or nascent DNA stability. (A) Immunofluorescence of RETSAT in PANC-1 cells with or without 0.2% Triton X-100 pre-wash ahead of paraformaldehyde fixation. (B) Co-immunostaining of RETSAT and telomeric PNA probe in BxPC-3 cells. (C, D) Co-immunostaining of RETSAT (green) and BrdU pulse labeled replication foci (red) in BxPC-3 and PANC-1 cells cultured under vehicle or HU induced stress conditions. (E) Co-immunostaining of RETSAT (green) and BrdU pulse labeled replication foci (red) in BxPC-3 cells under 21% or 0.3% O2 conditions. (F, G) Immunoblotting of RETSAT in PANC-1 cells treated with 4 mM HU (F) or 10 μM gemcitabine (G) at indicated time points. β actin was used as a loading control. (H, I) Experimental [file 13046_2022_2490_MOESM1_ESM.docx]
